# CDC (Cindy and David’s Conversations) game: Advising President to survive pandemic

**DOI:** 10.1016/j.isci.2023.107079

**Published:** 2023-06-09

**Authors:** Zhanshan (Sam) Ma, Liexun Yang

**Affiliations:** 1 Computational Biology and Medical Ecology Lab, State Key Laboratory of Genetic Resources and Evolution, Kunming Institute of Zoology, Chinese Academy of Sciences, Kunming 650223, China; 2 Center for Excellence in Animal Evolution and Genetics, Chinese Academy of Sciences, Kunming 650223 China; 3 Bureau of Planning and Policy, National Natural Science Foundation of China, Beijing 100085, China

**Keywords:** Public health, Information system model, Decision science

## Abstract

Ongoing debates on anti-COVID19 policies have been focused on coexistence-with versus zero-out (virus) strategies, which can be simplified as “always open (AO)” versus “always closed (AC).” We postulate that a middle ground, dubbed LOHC (low-risk-open and high-risk-closed), is likely favorable, precluding obviously irrational HOLC (high-risk-open and low-risk-closed). From a meta-strategy perspective, these four policies cover the full spectrum of anti-pandemic policies. By emulating the reality of anti-pandemic policies today, the study aims to identify possible cognitive gaps and traps by harnessing the power of evolutionary game-theoretic analysis and simulations, which suggest that (1) AO and AC seem to be “high-probability” events (0.412–0.533); (2) counter-intuitively, the middle ground—LOHC—seems to be small-probability event (0.053), possibly mirroring its wide adoptions but broad failures. Besides devising specific policies, an equally important challenge seems to deal with often hardly avoidable policy transitions along the process from emergence, epidemic, through pandemic, to endemic state.

## Highlights

• The world has been debating anti-COVID policies virtually from day one of the pandemic
• We model the problem as an evolutionary game dubbed CDC (Cindy & David’s Conversations)
• 16 strategies are possible but about half are of no policy values because of unsustainability
• Coexistence/zero-out/middle-ground (low-risk open high closed) should be stage-dependent

## Graphical abstract

**Figure:**
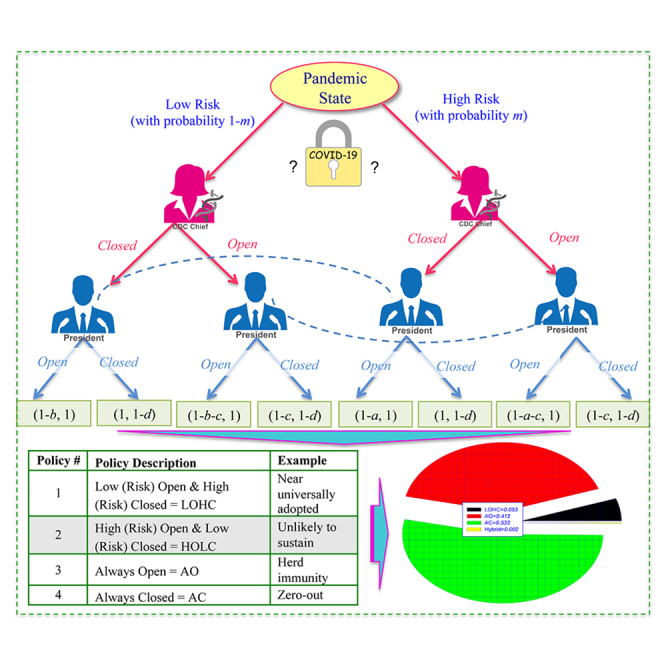

## Introduction—Cindy and David’s Conversations

*During the Covid-19 response, the greatest challenge to public health in more than 100 years,****science****must guide public health decision making.*

Sonja Rasmussen & Denise Jamieson (2020)^1^

[**Science**] *can inform policy, but it can’t dictate how to weigh the moral and political nature of policy makers’ decisions.*

Nason Maani & Sandro Galea (2021)^2^

*Human behavior evolves as our knowledge increases, but we are all subject to our own ways of construing this knowledge. Because of the pervasive influence of social media, new knowledge is often overwhelmed by misinformation that further confuses us and provides easy access to conspiracy theories and alternative facts.*

Nicholas Dirks (2021)^3^

*Another recurring theme in historical analyses of epidemics is that medical and public health interventions often fail to live up to their promise.*

David Jones (2020)^4^

The COVID-19 pandemic has been pushing the debates on anti-pandemic policy-making to an unprecedented contentious level both nationally and internationally. Perhaps the only certainty in the short term would be no consensus because of the complex strategic interactions among scientific, socioeconomic and international political factors. Arguably, the focus of these strategic interactions can be abstracted as an advisory relationship between a country’s advisory body (e.g., CDC chief scientist) and state head (e.g., President). In an idealized world, the chief is supposed to fully grasp the pandemic status and be capable to provide sound scientific recommendations to the president in time, and the president is supposed to make decisions that benefit the country, if not the world, most. In reality, either chief or president may be faulty in understanding the state-of-the-pandemic and their communication channel may be “noisy”, which may lead to unexpected and/or undesirable consequences. “Faulty” can be misjudgment of pandemic risk, and “noisy” communication can be misinformation or miscommunication; fault and noise can be associated with either chief, president, or both.

In the present study, we formulate the CDC-chief versus President relationship as an evolutionary game to facilitate the analysis of their strategic interactions using a fictitious pair of players (Cindy as CDC chief, who represent the expertise authority of medical science, versus David as President, the head of the decision-making body in general), and the CDC (Cindy and David’s Conversations) game is apparently inspired by the classic Sir Philip Sydney (SPS) game and its extensions (Maynard-Smith 1991, Maynard-Smith & Harper 2003, Bergstrom & Lachmann 1997, 1998, Huttegger & Zollman 2010, Ma 2009, Ma et al. 2010, Ma & Krings 2011, Whitmeyer 2020, Ma & Zhang 2022).^5^^,^^6^^,^^7^^,^^8^^,^^9^^,^^10^^,^^11^^,^^12^^,^^13^^,^^14^ We further take inspirations from classic ecological theories of metapopulation dynamics (Levins 1969, Citron et al. 2021)^15^^,^^16^ and Allee effects (Allee 1932, Hilker et al. 2009),^17^^,^^18^ in which the worldwide pandemic of COVID-19 can be abstracted as a dynamic system of *metapopulation* (of infected populations) consisting of approximately 200 local (country or regional) *populations*. The local populations are connected through migration/dispersal (such as international travels and trade logistics) and collectively form the global metapopulation, that is, *metapopulaton* is a *population* of *local populations*. According to metapopulation and Allee effects theories, local extinctions (zero-outs) can be as common as local outbreaks. Such phenomena may not only explain the highly heterogeneous distribution patterns of local outbreaks of the COVID-19 pandemic, but also shed lights on the intricacies of various anti-pandemic policies.

**Figure 1:**
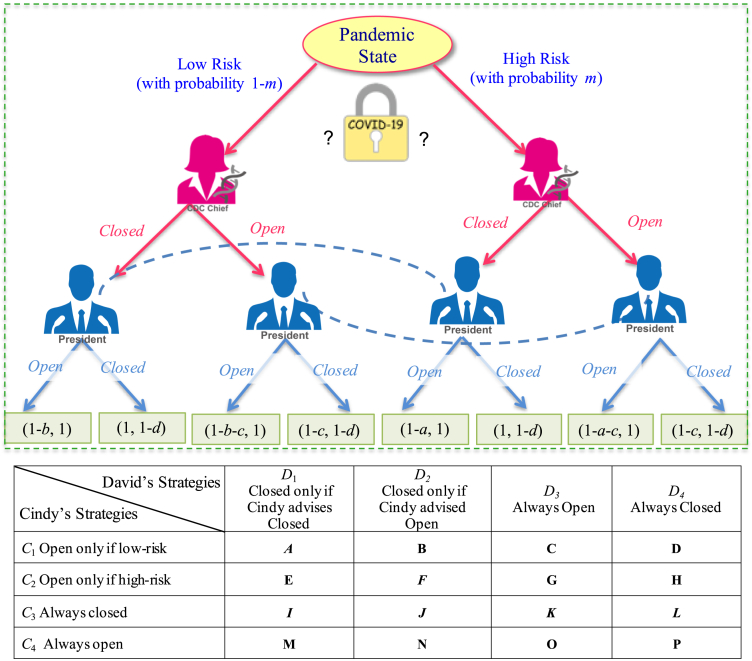
A decision-tree form of the CDC (Cindy and David’s Conversations) game formulated as a four-by-four asymmetrical SPS (Sir Philip Sidney) game: The terminal nodes show the payoffs (inclusive fitness) of Cindy and David, respectively The dotted lines depict Cindy’s information sets, but David may not be able to distinguish the decision nodes connected by the dotted lines. The bottom table illustrates the 16 (4x4) strategy pairs (A-P) of the CDC game, among which the *italic* strategies (*A, F, I, J, K, L*) are of equilibriums. Other equilibriums include polymorphic hybrid equilibriums (*PHE*), which are not shown here. *See*Table 1, STAR Methods (Box 1 and 2) for additional information on the formulation of the CDC game.

The CDC game is abstracted as an asymmetric four-by-four strategic-form game, 16 strategic interactions (Table 1, Figure 1, Box 1 and 2). The justifications why we adopted evolutionary game modeling, specifically the SPS game, have high significances to do with the five unique, fundamental characteristics of the SPS evolutionary game, as exposed below. Before presenting the five characteristics, we argue for a general strategic principle, which maintains that three fundamental processes or forces—competition, cooperation and communication (or 3C)—drive the biological (social) evolutions, including the policy-making for anti-pandemic. We do not argue that both biological (organismal) evolution and social evolution (development) are the same, and we only argue that the tools to study organismal evolution can be harnessed to study questions in social evolution because the 3C processes (forces) are major driving forces for their evolution (development) in both systems. Darwin’s evolutionary theory focused on competition and struggling for living, but largely ignored cooperation, not to mention communication that we argue is able to modulate competition and cooperation. In the 1960s, the critical significance of *cooperation* in the form of kin selection was introduced to supplement Darwin’s evolutionary theory (Hamilton 1964).^19^ Since then, five major forms of cooperation have been identified and most notably (Axelrod & Hamilton 1981, Nowak 2006, Nowak & Sigmund 2007)^20^^,^^21^^,^^22^, Prisoner’s dilemma (PD) was introduced and recognized as one of the most powerful models for modeling cooperation in evolutionary biology and social-evolution studies (Aumann 1959, Amadae 2016, Tanimoto 2015, 2021).^23^^,^^24^^,^^25^^,^^26^ The handicap principle first proposed in the 1990s filled the gap left by the evolutionary theory (Zahavi 1975, 1997, Grafen 1990, Maynard-Smith 1991)^5^^,^^27^^,^^28^^,^^29^, and its theoretical incarnation spawned the invention of the Sir Philip Sydney (SPS) game (Maynard-Smith 1991, Maynard-Smith & Harper 2003, Huttegger & Zollman 2010, Brown 2016, Cooper et al. 2018, Whitmeyer 2020, Ma & Zhang 2022, Zahavi 1975, 1997).^5^^,^^6^^,^^9^^,^^13^^,^^14^^,^^27^,^28^,^30^,^31^ In fact, evolutionary game theory (including SPS game model) was an integration of game theory and evolutionary theory (Maynard-Smith & Price 1973, Grafen 1990, Amadae 2016, Ma 2009, Ma et al. 2010, Ma & Krings 2011)^10^^,^^11^^,^^12^^,^^24^,^29^,^32^ and the integration turned evolutionary game theory into one of the most active branches of game theory.

**Table 1:** From Sir Philip Sidney (SPS) game to Cindy and David’s Conversations (CDC) game

**The Sir Philip Sydney (SPS) Game and Its Extensions (Maynard-Smith 1991, Huttegger & Zollman 2010, Ma & Krings 2011, Whitmeyer 2020, Ma & Zhang 2022)**^5^,^9^^,^^12^^,^^13^^,^^14^	

**Sender (Signaler) Strategies**	**Responder (Donor) Strategies**
*S*_*1*_: Signal only if healthy	*R*_*1*_: Donate only if no signal
*S*_*2*_: Signal only if needy	*R*_*2*_: Donate only if signal
*S*_*3*_: Never signal	*R*_*3*_: Never donate
*S*_*4*_: Always signal	*R*_*4*_: Always donate

**Cindy and David’s Conversations (CDC) game**	

**Cindy’s Strategies (Advices)**	**David’s Strategies (Decisions)**
*C*_*1*_: Open only if low-risk	*D*_*1*_: Closed only if Cindy advises closed
*C*_*2*_: Open only if high-risk	*D*_*2*_: Closed only if Cindy advises open
*C*_*3*_: Always closed	*D*_*3*_: Always open
*C*_*4*_: Always open	*D*_*4*_: Always closed
For examples: *C*_*1*_ is intuitively equivalent to: *Open* = When low-risk with probability (1–*m*); *Closed* = When high-risk with probability (*m*).	For examples: *D*_*1*_ is intuitively equivalent to: *Closed* = Following Cindy’s advice of closed with potential loss of *d* (such as dissatisfaction of public); *Open* = following Cindy’s advice of open without potential loss of *d*.

The 4x4 combinatorial asymmetric strategies of the discrete CDC game with the letters (A-P) for possible strategy pairs as shown in Figure 1				
Sender strategies (Cindy’s Advices)	Responder’s strategies (David’s decisions)			
	*D*_*1*_ Closed only if Cindy advises Closed	*D*_*2*_ Closed only if Cindy advises Open	*D*_*3*_ Always Open	*D*_*4*_ Always Closed
*C*_*1*_: Open only if low-risk	*A*	B	C	D
*C*_*2*_: Open only if high-risk	E	*F*	G	H
*C*_*3*_: Always closed	*I*	*J*	*K*	*L*
*C*_*4*_: Always open	M	N	O	P

Four Basic Anti-Pandemic Policies, which should be the output from the previous CDC game model (*see*Table 2 for the corresponding CDC equilibriums)			
Policy #	Policy Description	Possible CDC equilibriums likely to output the policies	Example
1	Low (Risk) Open & High (Risk) Closed = LOHC	Strategy *A*, Nash Equilibrium; Strategy *F*, ESS (evolutionarily stable strategies); Polymorphic Hybrid Equilibrium (*PHE*)	Near universally adopted
2	High (Risk) Open & Low (Risk) Closed = HOLC	No equilibriums possible; but B & E as non-equilibrium strategies are possible.	Unlikely to sustain
3	Always Open = AO	Mixed Strategy *J* & *K*, Pooling Equilibrium; *PHE*	Herd immunity
4	Always Closed = AC	Mixed Strategy *I* & *L*; Strategy *L*, Pooling Equilibrium; *PHE*	Zero-out

Box 1 **The three key elements (Who, What and How) to formulate the CDC game, the transformation from SPS through to the CDC game, and the interpretations of CDC Parameters**

**Table:** 

**Three key elements (*Who*, *What* and *How*) to formulate the CDC game and the exposition of the transformation from SPS through to the CDC game**	

(*i*) *Who* are the game players	*Sender* (Cindy or Adviser) vs. *Responder* (David or Decision-maker). *Sender* (signaler) was Sir Philip’s comrade, and *Responder* was Sir Philip Sydney in Maynard-Smith (1991) original SPS game. In the CDC game, Cindy is the message *sender* or *adviser*, and David is the *responder* or *decision-maker*. The essentiality of the role assignment lies in that who signals and who responds.
(*ii*) *What* is the content of message?	It is apparently the “request for water bottle” for the message *sender*, and the *responder* could either honor or ignore the request (water bottle) in the classic SPS game. The essentiality here is the message of “state-of-the-risk” (injured in SPS or high-risk in CDC) with probability of *m*.
(*iii*) *How* is message sent (signaled)?	Through shouting or whispering in classic SPS game. In the CDC game, it is through some communication channel such as phone, person conversation, or media. However, the ‘*How*’ point does not matter much in the sense that it does not influence the *mathematical logic* of the game.
4 (2x2)—Strategy Set in Classic SPS Game	There were only *four* (2x2) strategies in classic (Maynard-Smith 1991) SPS game since it was considered sufficient for its initial design, *i.e.,* to demonstrate the *handicap* principle or the *reliability* (*honesty*) of animal communications (Zahavi 1975, 1997). The initial hypothesis of the handicap principle was that to communicate honestly, there must be a handicap in the communication system that enforces the honesty. With the handicap principle, only four strategies that make sense. We use the term “make sense” to imply that the strategies are broader than being “rational” since evolutionary game theory does not enforce “rationality.” Instead, as long as there are ESS (evolutionary stable strategy) equilibriums, the strategies make sense. Actually, the strategies in the original SPS game not only sensible (natural), but also are rational, including sending dishonest signal (selfish behavior) since payoff was high for doing so.
16(4x4)-Strategy Set in the CDC Game	It was Huttegger & Zollman (2010), Whitmeyer (2020) and *etc*, which significantly expanded the classic SPS game, most notably, expanding the strategies from 4 to 16, which transformed the asymmetric, classic SPS to an *equivalently* reciprocal game given virtually all possible strategies are allowed. This extension allowed Ma & Zhang (2021) to describe various idiosyncrasies in masking behaviors in the context of ABD (Alice and Bob’s dating dilemma) game. Table 1 and Figure 1 listed the 16 possible strategies of the CDC game. The *strategy matrix* starts with (C_1_, D_1_) strategy: Open only if low-risk; D_1_: Closed only if Cindy advises closed. This is arguably one of the most rational “conversations” (advice-decision) in the CDC game. Once the first strategy pair (C_1_, D_1_) is specified, the remaining 15 pairs are set to keep consistent mathematic logics, as well as reciprocal interactions. The logic consistency refers to the validity of mathematic logic, which determines the rigorous inferences of the various equilibriums listed in Table 2. The equilibriums indicate that the corresponding strategies can be evolved and become established (adopted) in a society. Nevertheless, the validity of the mathematic logic does not guarantee the rationality or naturalness of strategies. That is, valid strategies can be senseless, mirroring various idiosyncratic decisions during the pandemic.
Potentially Alternative Strategy Matrices (Sets) for CDC game	Before finalizing the CDC strategy matrix as exhibited by Table 1 and Figure 1, we tried several alternatives. We realized that, after elements (*i: Who*) & (*ii: What*) are specified, the setting of (*iii: How*) does not matter in the sense that the setting only affects the *order* of the 16 combinatorial (interaction) strategies. It influences neither the validity of various strategy *equilibriums* (Table 2) nor the *completeness* (Table 1 or Figure 1) of the strategy set (options). That is, whatever order is specified, all possible strategy options (behaviors) are covered by the game (*i.e*., completeness). That is, although the classic SPS (with 4 strategy pairs) is not reciprocal, in the extended SPS game including CDC game, when the 12 ignored strategy pairs (due to the non-reciprocal or asymmetrical nature of SPS) were brought back. In effects, the CDC games cover all possible strategy options (*completeness*), and the strategic interactions are reciprocal due to the completeness.

**Interpretations of the parameters (*a*, *b*, *c*, *d*, *m* & *k*) in the SPS/CDC Games**	

*a, b, c, d*	Parameters *a* and *b* the cost parameters associated with Cindy, with *a* is the cost of misjudgment of high risk (underestimate risk), and *b* of low risk (overestimate risk), respectively. Parameter *c* is the strategic cost from the mistake in the CDC system. Parameter *d* is the cost parameter associated with David’s decision-making such as the decline of public support.
*m* & *k*	Parameter *m* and *k* are different from the previous previously defined parameters *a*, *b*, *c*, and *d* in the sense that they may influence ‘cost’, but they are not ‘cost’ *per se*. Parameter *m* is the risk level Cindy judges in a country (region), and parameter *k* is the relatedness between Cindy and David. Parameter *k* represents their shared interests (shared genes in evolutionary biology) and it has a direct impact on their *inclusive fitness* (*payoff*) (=*k* times the fitness of the other player plus his or her own fitness).

Box 2 **Definitions for the equilibrium types and stability properties in the SPS/CDC games**

**Table:** 

**Definitions for the equilibrium types**	

Nash equilibrium	A *Nash equilibrium* (*i.e.,* “no-regret” strategies) is a solution to a non-cooperative game involving two or more players, in which each player is supposed to be aware of the equilibrium strategies of the other players, and no player is better off by unilaterally changing own strategy. In other words, when each player has selected a strategy and no player may benefit from changing strategies whereas the other payers keep theirs fixed, then the current set of strategies and the consequent payoffs (fitness) constitute a Nash equilibrium.
ESS (evolutionarily stable strategy)	An *evolutionarily stable strategy* (ESS) is a strategy that, if adopted by a population of players in a given environment, cannot be invaded by any mutational (alternative) strategy, which is usually scarce initially. It is a Nash equilibrium that is “evolutionarily stable or resilient.” In other words, an ESS is a strong version of the Nash equilibrium, and the Nash equilibrium is a weak version of ESS.
Pooling equilibrium	A *pooling equilibrium* is an equilibrium in which, regardless of the types of sender with differing characteristic signals, the receiver shall choose the same response. In the case of the CDC game, David does not care what Cindy advises when he makes the decision.
Separating equilibrium	A *separating equilibrium* is opposite to pooling equilibrium: a receiver’s response can be different depending on the type of sender’s signal. In the case of CDC game, Cindy’s advice does influence David’s decision.
Hybrid equilibrium	A *hybrid equilibrium* is some combination of *separating* equilibrium and *pooling* equilibrium.
Polymorphism	A *polymorphism* is a mixed Nash equilibrium where sender mixes between being honest and dishonest signaling, and responder may also adopt a mixed strategy accordingly. Polymorphisms, which were ignored in biological signally until Huttegger & Zollman (2010) extensions of the original SPS game, correspond to hybrid equilibriums in this study. Polymorphic equilibrium allows for partially meaningful (honest) communication, and therefore grants the SPS much realism.

**Definitions for the stability properties**	

Lyapunov Stable	Assume a dynamical system which is described with $\dot{x}=f\left(x\right),\begin{array}{cc} & x\in R^{n} \end{array}\text{,}$ there is an equilibrium solution $\bar{x}\in R^{n}$, such that $f\left(\bar{x}\right)=0$. The equilibrium solution $\bar{x}$ is stable in the sense of Lyapunov stable, if for given ε>0, there exists δ=δ(ε)>0 such that, for any other solution y(t), $\|y\left(t_{0}\right)-\bar{x}\|<\delta \;\begin{array}{c} \left(t_{0}\in R\right) \end{array}$, then $\|y\left(t\right)-\bar{x}\|<\varepsilon$ for $t>t_{0}$. Lyapunov stability is a rather weak requirement on equilibrium points. Especially, it does not require that trajectories starting close to the origin approach to the origin asymptotically. In a simplified interpretation, if the solutions that start out near an equilibrium point *x* forever, then *x* is Lyapunov stable.
Asymptotically Stable	A time-invariant system is asymptotically stable if all the eigen-values of its system matrix (A) possess negative real parts. In the case of asymptotic stability, there is a sphere *S*, centered around *δ* = 0 with the radius *r*, such that the response, once entered the sphere, converges to the origin.
Neutrally Stable	An equilibrium that is Lyapunov stable but not asymptotically stable is sometimes termed as *neutrally stable*.

First, SPS game was invented by Maynard-Smith (1991) to model the reliability (honesty) of animal communication by narrating a well-known story about British poet-soldier Sir Philip Sidney (1554–1586), who was fatally injured in a battle, with the immortal words “*thy necessity is greater than mine*”, passed his water bottle to one fellow soldier who was also a casualty.^5^ Somewhat ironically, when transforming the story into the SPS game, Maynard-Smith (1991) introduced dishonest (cheating) possibilities in this heroic legend: Sir Philip’s comrade could cheat, i.e., requesting a water bottle while he was not actually injured.^5^ On the other hand, Sir Philip could be selfless by donating the water bottle to his comrade or selfish by doing nothing. The selfish versus selfless and dishonest versus honest essentially introduce the cooperation and communication into SPS game, respectively. Because, to some extent, competition and cooperation can be considered as both sides of same coin, and communication can alter balance between competition and cooperation, or modulate the both, therefore, the SPS game spans the whole 3C processes (forces).

Second, SPS game was invented to demonstrate the handicap principle, which states that the signal of animal communication must be costly to be reliable (honest) and there is a strategic cost (the so-termed handicap) that ensures the honesty. For example, the peacock tail in flight is considered as a classic example of a handicapped signal of male quality, which signals the superior fitness of males to females in sexual selection. In human society, luxurious goods must be costly to be valuable (signaling social status). In the case of the CDC game, there is a strategic cost (*c*) associated with miscommunication between Cindy and David. The SPS game not only provides a framework to model the strategic cost (*c*) capturing the possible noise communication between Cindy and David, but also offers measures to capture the costs associated with Cindy and David. For Cindy, there are two cost parameters (*a, b*) that are associated with underestimating or overestimating pandemic risk (*m*) level. For David, there is a cost parameter (*d*) that can capture the cost associated with David’s possible misjudgment. In short, SPS game and more accurately extended SPS game model have four cost parameters (*a, b, c, d*) that can capture the costs associated with their potential faults and their miscommunications. For strategic modeling, this structure of cost parameters is sufficiently specific to capture major cost determinants but general enough for inferring equilibrium analytically.

Third, the extended SPS game has a relatedness parameter *k*, which is termed inclusive fitness (payoff) and measures the cooperation tendency between two game players. This fifth parameter (*k*) of CDC game is hence designed to measure cooperation (or competition) tendency in the policy-making system.

Fourth, as mentioned previously, there is a sixth parameter—the pandemic risk (*m*), which is the counterpart of injury probability of Sir Philip’s comrade in the classic SPS game. With the above-explained six parameters, the proposed CDC game is able to capture the estimation of pandemic *risk* (*m*), the *costs* (*a, b, c, d*) associated with reliability of the estimation, possible faults by Cindy and David, *benefits* (payoff) of the game strategies (analytically derived from the CDC game model). Finally, we have the relatedness parameter (*k*) that captures the intricacies of the strategic interactions between CDC chief and president. Although the president may ignore the chief’s advice for political consideration, they do have shared interests measured by *k*. With the six parameters, a system of 8 differential equations is formulated to describe the dynamics of the CDC game, and possible equilibriums of the dynamic systems for 16 strategy interactions can be analytically obtained and simulations can be performed. Using differential equations to formulate games as dynamic system rather than the traditional matrix model is a huge advantage of the evolutionary game, and allows for obtaining evolutionary stable strategies, which means that “mutation” of strategies with time are considered, and the strategies should be sustainable over time in general from a practical perspective.

Fifth, the SPS has an advantage over the familiar PD game, namely it can simultaneously capture all three fundamental processes (forces) driving biological and/or social evolution, whereas the PD game can only capture two (competition and cooperation) excluding communication. For example, in the PD game, there are no direct communications between the two players (criminals) and they are questioned by police officer separately. In the SPS game, both players have explicit communications, and in fact, the game is a request-response game. Furthermore, the communicated information can be asymmetrical (deception). This difference makes SPS game a more appropriate choice than PD game for our research purpose.

Intuitively, and also in fact, there are arguable only four major kinds of anti-pandemic, public-health *policies* regarding lockdown or reopen, including (1) low-risk open & high-risk closed (LOHC), (2) high-risk open & low-risk closed (HOLC), (3) always open (AO), and (4) and always closed (AC). These are four fundamental *policies* or practices a country (region) may adopt and, in this article, we distinguish *policies* (referring to the above four) from *strategies*. Note that although we use the term policy in this article because we believe the usage is more natural in the context of public-health policy-making, the four policies described above and referred to in later sections, strictly speaking, should be termed strategy or meta-strategy in the terminology of game theory.

With the CDC game, we postulate that the four policies should be mapped to the output of the CDC game system, specifically, the ultimate decisions *made* by David with advice from Cindy. In other words, policies are the product or output of the decision-making process (system) such as the strategic CDC game. By distinguishing between the policy and strategy, one important gain is to shed light on the mechanism or process of decision-making process—how a particular policy can be reached by the advisor-decision-maker body such as CDC-President taskforce. Furthermore, one can also investigate the influences of various pandemic and/or socio-economic factors (as captured by the previous mentioned six parameters) on the policy-making process and outcome.

We admit that the *four-policy* classification is the result of somewhat simplified view, which is necessary as explained previously; nonetheless we argue that it is sufficiently general to cover the full spectrum of currently adopted anti-COVID policies in the world. At present, the LOHC policy is adopted by most countries (regions) in the world, whereas no country has adopted its opposite (i.e., HOLC), which is irrational if not anti-intelligent. A handful of countries have adopted *laissez-faire* strategy (passive herd immunity), which is equivalent to AO policy. The strict AC policy appears impractical and no country (region) has claimed to adopt such a policy. However, arguably, the dynamic zero-out (infections) adopted by a handful of countries such as China and New Zealand may be considered as either AC (in terms of effects) or LOHC (in terms of implementation).

## Results–The CDC game model

### The conceptual formulation of CDC game model

After near two years of struggling against the COVID-19 pandemic, the vaccinations are bringing up, but the delta and emerging lambda are damping down, the hopes for reopening. Cindy, the CDC (center for disease control) chief scientist, is preparing her weekly briefing for the president, David, a newly elected president who has campaigned to bring peace and super humanity to the country and world. Cindy’s team has been evaluating the nation’s status of pandemic and vaccinations, and she realized that her team can only obtain a simple probability metric (*m*) to deliver her evaluation of the pandemic status, with *m* being worsen (high risk) and (1-*m*) alleviated (low risk). David, on the other hand, may need to balance the health versus wealth, the public interests versus other potentially conflicting interests. He may or may not follow Cindy’s advice with potential loss (*d*) depending on the reliability of Cindy’s advice and other factors on his side such as potential influences on economy and political correctness of his decision.

Furthermore, the trust between Cindy and David has been raising red flag, and their cooperative relationship is far from perfect. For example, given that Cindy was appointed by a previous administration and she might also be concerned with the risk of becoming a scapegoat in the case of catastrophic failure in anti-pandemic policy. In addition, Cindy’s aptitude may be imperfect and can be faulty. In extreme cases, Cindy may delay or even hide her evaluation of the true state-of-the-pandemic. Cindy’s potential faults, errors (benign faults) or dishonesty (malicious faults) can carry potential loss (cost) in the form of parameter *a* (underestimating risk *m*) or *b* (overestimate risk *m*), which influences her fitness or payoff.

Despite the potential communication issues between Cindy and David, both of them are certainly patriots and share common interests, and they tend to collaborate closely to maximize public and national interests. However, Cindy’s care of her reputation as a scientist and David’s care of his public approval rates are not necessarily always consistent, and may create competition. The balance between cooperation and competition between their interests can be measured by a *relatedness* parameter *k,* which is proportional to their tendency of cooperation. Furthermore, there is the cost (*c*) associated with strategic mistakes, the medical and socioeconomic consequences of misjudgment or miscommunication of the true state of pandemic, including consequences of possible cascading faults from both Cindy and David, i.e., cost of systemic failure.

Table 1 and Figure 1 exposed the formulation of the CDC (Cindy and David’s Conversations) problem as an extended SPS (Sir Philip Sydney) game. Box 1 further exposed the three key elements (Who, What, and How) to formulate the CDC game, the transformation from the SPS through to the CDC game, as well as the interpretations of the CDC parameters. The so-termed extended SPS game refers to the transformation of the classic SPS game (Maynard-Smith 1991)^5^ from 2x2 to 4x4 asymmetrical game performed by Bergstrom & Lachmann (1997, 1998), Huttegger & Zollman (2010), Whitmeyer (2020), *etc.*^7^^,^^8^^,^^9^^,^^14^ In Table 1, the top section is the Sir Philip Sidney (SPS) game proposed by Maynard-Smith (1991)^5^ and further extended by Huttegger & Zollman (2010)^9^, and Whitmeyer (2020)^14^, as well as Ma & Zhang’s (2022)^13^ ABD game. In formulating the CDC game (the bottom section), we adopted slightly different symbols with the classic SPS game, with Cindy for advisor (signaler or message sender) and David for decision-maker (responder or message receiver). For the CDC game, Table 1 listed the 4 possible options (strategies) Cindy and David each may have and their 16 (4x4) possible strategy interactions, mirrored 16 options of the CDC game or the 16 elements (cells) of the strategy matrix. Figure 1 further illustrates the CDC as a decision-tree and strategy matrix. The differential equation system for the CDC game is introduced in the next sub-section.

As exposed in Table 1, Figure 1, and Box 1, with the strategy *C*_*1*_, we map “open” (low risk) in the CDC to signaling (healthy) in the classic SPS, i.e., *C*_1_ = “Cindy advises open only if she judges low-risk.” This is equivalent to map “closed” to “high risk.” Regarding David’s response, we map “closed” (high risk) in the CDC to “no signaling” in the original SPS, i.e., *D*_*1*_ = “Closed only if Cindy advises closed.” Other strategy interactions can be mapped similarly.

As further exposed in Table 1 and Box 1, there are three elements in *trans*-formulating the classic SPS into CDC game, *who* (are the game players), *what* (messages are sent and responded), and *how* (the message is transmitted). From the classic SPS to the extended SPS games including our CDC game, the strategy set is expanded to 16 (4x4) strategy interactions from 4 (2x2) interactions. Besides the three elements, a few additional points are particularly worthy of reiterations (see Box 1 for the details). First, once the first cell (*C*_*1*_, *D*_*1*_) in the strategy matrix (set) is specified, the remaining 15 cells are set to keep consistent mathematic logic. Actually, once the “*who*” and “*what*” are set, the third element (“*how*”) may only influence the order of strategies in the strategic matrix, but not their mathematical logic. Second, although the classic SPS game is 2x2 asymmetric and not reciprocal, the extended SPS game including the CDC game is, in effects, equivalent to a *reciprocal* game given that virtually all 4x4 (16) possible strategy options are included in the strategy set (completeness). Owing to this completeness or reciprocal equivalence, approximately 1/2 of the strategies may be unnatural or senseless (without discernible meaning or purpose), if the other 1/2 is considered as natural or rational. We take the advantages of those unnatural strategy interactions to capture the idiosyncrasies in decision-making for anti-pandemic, some of which could be not only counter-intuitive, but also unscientific, mirroring the various faults/noises in CDC explained previously.

In Table 1 and Figure 1, a series of 16 labels (A-P) for the 4x4 combinatorial strategies (strategy pairs or strategic interactions) of Cindy and David are assigned to facilitate the discussion on the CDC game. Furthermore, the bottom section of Table 1 also list the four basic anti-pandemic policies introduced in the introduction section, and their corresponding equilibriums in the CDC game, but further explanation of the relationship between policies and equilibriums are delayed to the section of results. Note that we use the letters (A-P) in bold font to denote the strategy pairs, which are the combinations of Cindy and David’s strategies that are denoted with *italic* letters (*C*_*1*_*-C*_*4*_, *D*_*1*_*-D*_*4*_). In addition, we use the *bold italic* fonts to denote those strategy pairs (out of the 16 possible pairs of A-P) with equilibriums (e.g., *A*, *F*). Strictly speaking, A-P represent 16 strategy pairs, i.e., the combinatorial strategies of Cindy and David, each of whom has 4 strategies (*C*_*1*_*-C*_*4*_, *D*_*1*_*-D*_*4*_). In the following, whenever no confusion arises, we may call the strategy pairs (A-P) strategy for short. These conventions of letter usages are followed throughout the article.

For the mathematical model (systems of differential equations) of the CDC game model as well as the detailed parameter interpretations (designations), refers to the methods detail in the STAR Methods section.

### Equilibriums and stabilities of the CDC game

In this section, we take advantage of Huttegger & Zollman (2010)^9^ extensions and analytical results on the extended SPS game, and summarize the equilibriums of the CDC game in Table 2, and the payoff of the CDC game strategies in Table 3, respectively. From Equations 1 and 2, six types of equilibriums are derived and summarized in Table 2, and a brief description is presented below.

**Table 2:** From CDC (Cindy and David’s conversations) game equilibriums/stability to anti-pandemic policies

Equilibrium Type (*see*Box 2 for definitions)^b^	Necessary conditions for the existence of the equilibriums (*see*Box 1 for CDC parameters)	Stability Property (*see*Box 2 for the definitions)^b^	Anti-pandemic Policy (also *see*Table 1)
*A*—Nash equilibrium^b^ (Apparently rational equilibrium)	a>kd−c>b & a>(d/k)>b	Asymptotically stable	LOHC (low-risk open and high-risk closed)
*F*—Signaling ESS^b^ (Separating equilibrium)	a>c+kd>b & a>(d/k)>b	ESS (Evolutionary Stable Strategy) and is Asymptotically stable.	LOHC
a*J* and *K* are behaviorally equivalent. $\left(C_{3},\left(1-\lambda \right)D_{2}+\lambda D_{3}\right)$ —Pooling equilibrium	d>k[ma+(1−m)b] & λ≥1−[c/(a−kd)]	Neutrally stable	AO (always open)
a*I and L* are behaviorally equivalent. $\left(C_{3},\left(1-\mu \right)D_{1}+\mu D_{4}\right)$ —Pooling equilibrium^b^	d<k[ma+(1−m)b] & μ≥[1−c/(kd−b)]	Neutrally stable	AC (always closed)
*L*—Pooling equilibrium	d<k[ma+(1−m)b]	Neutrally stable	AC
$\left(\lambda C_{2}+\left(1-\lambda \right)C_{4},\mu D_{2}+\left(1-\mu \right)D_{3}\right)$ —bPolymorphic Hybrid equilibrium (*PHE*) $\lambda =\frac{k\left[ma+\left(1-m\right)b\right]-d}{\left(1-m\right)\left(kb-d\right)}$ & $\mu =\frac{c}{b-kd}$	a>(d/k)>b & b−kd>c & d>k[ma+(1−m)b]	Lyapunov stable (Asymptotically stable or unstable)	LOHC, AO, or AC

a *J* & *K* mean that (*C*_3_, *D*_2_) and (*C*_3_, *D*_3_) are behaviorally equivalent. Similarly, *I* & *L* mean that (*C*_3_, *D*_1_) and (*C*_3_, *D*_4_) are behaviorally equivalent.

b See Box 2 for the detailed explanations, here is brief but less precise description for the equilibrium types: Nash equilibrium = no-regret strategies; Pooling equilibrium = “dictatorship” style strategy interaction, David ignores advice; Separating = opposite to pooling. ESS (evolutionary stable strategy) = Stronger than Nash, evolutionarily consistent. Polymorphic hybrid equilibrium (*PHE*) = sometimes pooling and sometimes separating (exclusively either), and mixed Nash equilibriums.

**Table 3:** The expected payoffs for Cindy and David at the equilibriums of the CDC game

Strategy Pair	Cindy’s Payoff (Fitness)	David’s Payoff (Fitness)
*A* — (*C*_*1*_: Open only if low-risk; *D*_*1*_: Closed only if Cindy advises closed)	1−b−c+k+m(b+c−dk)	1+k(1−b−c)+m(kb+kc−d)
B — (*C*_*1*_: Open only if low-risk; *D*_*2*_: Closed only if Cindy advises open)	1−c+k(1−d)+m(−a+c+kd)	1+k(1−c)−d+m(−ak+ck+d)
C — (*C*_*1*_: Open only if low-risk; *D*_*3*_: Always open)	1−b−c+k+m(−a+b+c)	1+k(1−b−c)+mk(−a+b+c)
D — (*C*_*1*_: Open only if low-risk; *D*_*4*_: Always closed)	1−c+k(1−d)+mc	1−d+k(1−c)+mkc
E − (*C*_*2*_: Open only if high-risk; *D*_*1*_: Closed only if Cindy advises closed)	1+k(1−d)+m(−a−c+kd)	1−d+k+m(−ka−kc+d)
*F* — (*C*_*2*_: Open only if high-risk; *D*_*2*_: Closed only if Cindy advises open)	1−b+k+m(b−c−kd)	1−bk+k+m(bk−ck−d)
G — (*C*_*2*_: Open only if high-risk; *D*_*3*_: Always open)	1−b+k+m(−a+b−c)	1+k(1−b)+mk(−a+b−c)
H — (*C*_*2*_: Open only if high-risk; *D*_*4*_: Always closed)	1+k(1−d)−mc	1−d+k−mkc
*I* — (*C*_*3*_: Always closed; *D*_*1*_: Closed only if Cindy advises closed)	1+k(1−d)	1−d+k
*L* — (*C*_*3*_: Always closed; *D*_*4*_: Always closed)		
*J* — (*C*_*3*_: Always closed; *D*_*2*_: Closed only if Cindy advised open)	1−b+k+m(−a+b)	1+k(1−b)+mk(−a+b)
*K* — (*C*_*3*_: Always closed; *D*_*3*_: Always open)		
M — (*C*_*4*_: Always open; *D*_*1*_: Closed only if Cindy advises closed)	1−b−c+k+m(−a+b)	1+k(1−b−c)+mk(−a+b)
O — (*C*_*4*_: Always open; *D*_*3*_: Always open)		
N — (*C*_*4*_: Always open; *D*_*2*_: Closed only if Cindy advises open	1−c+k(1−d)	1−d+k(1−c)
P — (*C*_*4*_: Always open; *D*_*4*_: Always closed)		

#### Strategy A has a Nash equilibrium and can be mapped to LOHC (low-risk open and high-risk closed) policy

With the strategy (pair or interaction) *A* of (*C*_1_ versus *D*_1_), Cindy advises for *open* only if she perceives low risk of pandemic, and David decides *closed* only if Cindy advises for *closed*. In this strategic interaction, David follows Cindy’s advice, whereas Cindy seems rational with her strategy of “open only if low risk.” The strategy pair is Nash equilibrium and is asymptotically stable (*see* Box 2 for the terminology interpretations). Overall, the strategy pair *A* seems to be one of the most rational CDC strategy interactions from scientific perspective. Furthermore, because *A* is Nash equilibrium, nobody would regret for their decisions once they are committed. Strategy *A* appears to be the most natural and rational strategy.

Strategy *A* is nearly universally accepted by most countries in the world, and it is mapped to the number #1 anti-pandemic public health, i.e.*,* the low (risk) open and high (risk) closed (LOHC) (Tables 1 and 2). The LOHC is arguably the most rational among the four fundamental policies. The LOHC policy can be obviously made from strategy *A* with Nash equilibrium. However, the polymorphic hybrid strategy (*PHS*) can also lead to the LOHC policy, which may explain the idiosyncrasies occurred in various parts of the world regarding the anti-pandemic policy. That is, although the LOHC is adopted ultimately, the decision-making process to reach the policy could involve some irrational or unnatural strategy interactions, e.g., could be a trial-and-error process.

#### Strategy F is an ESS (evolutionary stable strategy), asymptotically stable, and can be mapped to LOHC policy

With the strategy pair *F* of (*C*_2_ versus *D*_2_), Cindy appears irrational and advises for open if the risk is high, and perhaps she believes that the cost for closure is too high even though the pandemic risk is high. David appears more rational and overrules (reverses) the advice from Cindy, possibly either he does not trust Cindy’s advice or he believes that the benefits from closure outweigh the cost when the risk is high. Because *F* is an ESS, their decisions (behaviors) may be mutated when the pandemic expands and, ultimately, they may be converted from one strategy to another. Also because *F* is asymptotically stable, their consensus (equilibrium) has a region (range), which means both Cindy and David have certain level of flexibility in their behaviors. Overall, in this strategy interaction, the president (David) seems to have an intuitive and more rational attitude to the risk and decision than his advisor, Cindy.

Specifically, when the risk is high, Cindy would advise for open per *C*_2_ strategy, but David would choose to close per D_2_ strategy (David reverses Cindy’s recommendation). Hence, the strategy *F* again maps to the LOHC policy, as strategy *A* does. When the risk is low, Cindy offers no advice (her default option), but David has to make a decision anyway. Without advice from Cindy, he would choose his default option of open. That is, when no advice from Cindy, there should be an alternative to the scenario when Cindy advises him. With this logic, David’s decision would still lead to rational LOHC policy. Therefore, the strategy interaction *F* can evolve to an evolutionary stable strategy, and be mapped to the LOHC policy ultimately.

Therefore, both strategy *A* and *F* interactions can generate the LOHC policy: in the case of *A* strategy interaction, David usually simply follows Cindy’s advice, whereas in the case of *F*, David usually overrule Cindy’s advice.

#### Strategy L is a pooling equilibrium and can be mapped to AC (always closed) policy

With the strategy pair *L* of (*C*_3_ versus *D*_4_), Cindy always advises closure regardless of the risk level, and David always decides closure regardless of Cindy’s advice. The equilibrium is neutrally stable. From a pure theoretic perspective, the equilibrium type *L* is more likely to become established because it has a larger basin of attraction, which measures the likelihood of evolving under standard evolutionary dynamics (Equations 1 and 2). From a pure health perspective without considering wealth, the strategy can be optimum. Overall, the strategy pair appears to mirror a pair of advisor and decision-maker who take totally same attitudes to the disease and risk. Because the equilibrium of strategy is a pooling equilibrium, Cindy’s has no influences on David although Cindy’s advice is always consistent with David’s decision.

Obviously, this strategy interaction can be mapped to AC (always closed) policy for anti-pandemic. This strategy may be an optimum if the pandemic is over within a reasonably short period or economic loss is not a concern. Furthermore, if pandemic can be suppressed in a short period, the economic loss would not be a concern either.

#### Mixed strategies of J and K are pooling strategies and both are behaviorally equivalent, and can be mapped to AO (always open) policy

When Cindy adopted strategy *C*_3_ (always closed), and David adopted a mixed strategy of *D*_2_ (Closed only if Cindy advises open) with probability (1−λ) and *D*_3_ (always open) with probability λ, there is a pooling equilibrium that is neutrally stable. The condition for the equilibrium to occur is d>k[ma+(1−m)b]. David’s mixed strategy is $\left(1-\lambda \right)D_{2}+\lambda D_{3}$, and λ≥[1−c/(a−kd)] determines the strategy of David.

Theoretically, when David hesitates, he can resort to λ to make an optimal decision, i.e., choose *D*_*2*_ when λ≥[1−c/(a−kd)], or *D*_*3*_ when λ<[1−c/(a−kd)]. Because Cindy always advises for closure, David would always decide to open. Strategies *J* and *K* are behaviorally equivalent with each other.

A scenario for achieving this equilibrium can be like this: Cindy always advises for closure regardless of the risk, David may hesitate but in the end he still ignores Cindy’s advice and keep open. This scenario maps to AO (always open) policy. Herd immunity strategy could be considered as belonging to this strategy. In addition, the equilibrium of mixed *J* & *K* strategies is pooling, which implies that David’s decision is ‘dictatorship’ style. That is, although he may hesitate, but ultimately still ignores Cindy’s advice.

#### Mixed strategies of I and L are pooling strategies and both are behaviorally equivalent, and can be mapped to AC (always closed) policy

When Cindy adopted strategy *C*_*3*_ (always closed) and David adopted a mixed strategy of *D*_*1*_ (Closed only if Cindy advises so) with probability (*1*−*μ*) and *D*_*4*_ (always closed) with probability μ, where μ=1−[c/(kd−b)], there is a pooling equilibrium that is neutrally stable. Similar to previous *J* & *K* strategies, there is a pooling equilibrium that is neutrally stable, but here *μ* determines the strategy of David. Intuitively, because Cindy always advises closed, David may hesitate: but in the end, he still decides to close. The condition to determine David’s behavior seems to be opposite with the previous mixed strategy interaction of *J* & *K*. Strategies *I* and *L* are behaviorally equivalent with each other.

In practice, similar to strategy *L*, this mixed strategy of *I* and *L* maps to AC (always closed). The policy should be optimum when pandemic ends in a short period (“wealth” is not a concern) or economic loss from closure is not a concern.

#### *Polymorphic hybrid strategies* (*PHS*)

Cindy adopted a mixed strategy of *C*_2_ (open only if high-risk) and *C*_4_ (always open), and David also took a mixed strategy of *D*_2_ (closed only if Cindy advises open) and *D*_3_ (always open). With these options, there are hybrid equilibriums that are *polymorphic*. Overall, Both Cindy and David may hesitate, but they seem partially rational in the sense that Cindy may consider risk level and David may follow Cindy’s advice. For Cindy, she may recommend open or closed, and for David he may decide open or closed depending on the conditions explained below. This obviously is the most sophisticated type of equilibrium.

First, how would Cindy make her recommendation? In fact, it is the parameter μ that determines her recommendation to choose either *C*_2_ or *C*_4_. Theoretically, when she hesitates, she can resort to μ to make an optimal decision, i.e., choose *C*_2_ when μ>c/(b−kd), *C*_4_ when μ<c/(b−kd). When μ=c/(b−kd), she tends to adopt mixed strategy $\lambda C_{2}+\left(1-\lambda \right)C_{4}$.

For example, when μ>c/(b−kd), Cindy would prefer to choose C_2_, she would advise open even if high risk. This choice would suggest that Cindy is skeptic of closure. In contrast, when μ<c/(b−kd), Cindy would advise open anyway.

**Figure 2:**
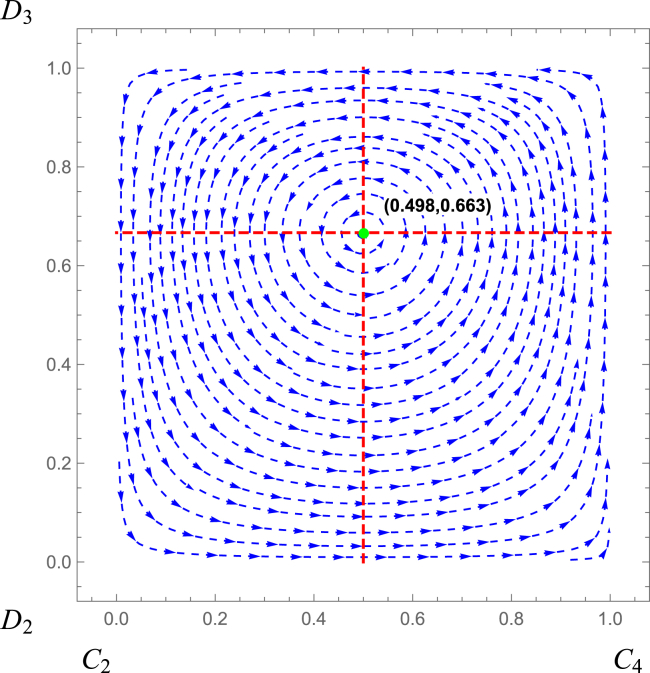
An example output from the simulation of the CDC game, i.e., the phase portrait of the polymorphic hybrid equilibrium (PHE) with the hybrid strategy of Cindy (*C*_2_, *C*_4_) versus David (*D*_2_, *D*_3_) under following parameter values: *a* = *0.7*, *b* = *0.3*, *c* = 0.1, *d* = 0.3, *m* = *0.6* and *k* = 0.5 Cindy and David make their decisions based on (*λ, μ*)=(0.498, 0.663) (the green circle). If *μ > 0.663,* Cindy prefers to choose *C*_*2*_ over *C*_*4*_, as indicated by the direction of portrait arrow (to left). If *μ < 0.663,* Cindy prefers to choose *C*_*4*_ over *C*_*2*_, as indicated by the direction of portrait (to right). When *μ = 0.663,* the *mixed strategy of C*_*2*_ and *C*_*4*_ is chosen. Similarly, David makes his strategic decisions based on the value of parameter *λ > 0.498, <0.498 or =0.498.*

Second, how would David make his decision of *D*_2_ or *D*_3_ in response to Cindy’s advice? David’s decision would depend on parameter λ. When $\lambda <\left\{K\left[ma+\left(1-m\right)b\right]-d\right\}/\left[\left(1-m\right)\left(kb-d\right)\right]$, David would prefer to choose D_2,_ (closed only if Cindy advises open, i.e., overrule or negate Cindy’s advice), or D_3_ (always open) when $\lambda >\left\{K\left[ma+\left(1-m\right)b\right]-d\right\}/\left[\left(1-m\right)\left(kb-d\right)\right]$. When λ={K[ma+(1−m)b]−d}/[(1−m)(kb−d)], he tends to adopt mixed strategy $\mu D_{2}+\left(1-\mu \right)D_{3}$. This hybrid equilibrium is Lyapunov stable, which implies that the actual stability may vary depending on the values of parameters. In other words, the stability of equilibriums may be broken when conditions such as risk level and/or cost change dramatically. Figure 2 shows an example of the decision-making in the phase space of the polymorphic hybrid equilibrium, obtained from the simulation study explained below. The polymorphic hybrid strategies, arguably, represent the most sophisticated options, which reflect the dynamic (evolutionary) nature of the masking-or-not decision-making.

In practice, the *polymorphic hybrid strategy* (*PHS*) may lead to three of the four policies, except for irrational HOLC (high-risk open & low-risk closed). For example, with (*C*_2_, *C*_4_) and (*D*_2_, *D*_3_) strategy interactions, any of the four strategy interactions (*F*, *G*, *N*, *O*) can happen. If *F* is chosen, then it may lead to LOHC; if *G* or *O* is chosen, then it may lead to AO; if *N* is chosen, it may lead to AC. Note that none of the polymorphic strategies with equilibriums can be mapped to HOLC, which indicates that the HOLC is so irrational and is unlikely to sustain (reaching equilibrium) in practice.

In summary, as listed in Table 1 (the bottom section) and Table 2 (the last column), arguably the most rational LOHC policy can be the output of equilibriums *A* & *F*; the AO policy can be the output of equilibriums *J* & *K*; the AC policy can be the output of equilibriums *I* & *L* or *L*. The polymorphic hybrid strategy (*PHS*) can produce any of the three previously discussed policies, i.e.*,* LOHC, AO, or AL. However, none of the six types of equilibriums can produce the HOLC policy, which should be expected that given the policy is unlikely to sustain in practice.

#### Strategy interactions without equilibriums

For strategy interactions that do not produce equilibriums, there are the following mappings: strategy interactions D, H, L, P, N, and *I* can produce AC policy, and strategy interactions C, G, K, O, M, and *J* can produce AO policy. Because these strategy interactions, except for *I*, *J*, and *K* do not have known equilibriums, which implies that they may not be sustainable in practice, we do not discuss them further in this study.

Finally, strategies B & E can be mapped to irrational HOLC policy, but both the strategies do not have any equilibrium (Table 2), which should be expected and is consistent with the reality that the HOLC policy is so irrational and is unlikely to sustain practically.

### Simulation experiments

#### Simulation procedures and phase portrait

Because the analytic solutions (Tables 2 and 3) are less intuitive in interpreting the decision-making process of the CDC game, particularly, in understanding the influences of various CDC parameters (cost/benefit factors, risk level, cooperation tendency), we performed simulation experiments based on the replicator dynamics (Equations 1 and 2). Tables S1, S2, S3 and S4 contain the 4 lists of equilibriums corresponding to the three public health policies (LOHC, AC, AO), and the list of *PHS*, which may be mapped to one of the previous three policies, depending on the specific parameter values of the CDC game. The simulations were performed based on the replicator dynamics (Equations 1 and 2) with a program developed by Ma & Zhang (2022).^13^ Specifically, the equilibriums were simulated and determined based on the necessary conditions (listed in Table 2) and corresponding payoffs were computed based on the equations listed in Table 3, with simulation step length = *0.1* for all of the model parameters (*a, b, c, d, k, m*), all of which range from *0* to *1*. A total of *10*^*8*^ simulations were performed to obtain the results, and the results were organized in terms of the policy category (online Tables S1, S2, S3 and S4).

The strength of simulations helps us to illustrate the complex interactions between advisor and decision-maker intuitively, but the simulations cannot be exhaustive because of the continuous nature of the CDC model. In the following, we use the simulations to primarily address two questions: (1) The influences of various CDC parameters on the outcome (policies) of CDC game; those parameters approximate the cost/benefits, risk level, faults, miscommunications (dishonesty), *etc* in the decision-making system. (2) Likelihood of each policy—the estimated probability of the policies based on *10*^*8*^ times of simulations. Before addressing the two questions, we demonstrate the simulation of *PHS* with a phase portrait (Figure 2) to illustrate possible interactions between Cindy and David in a hybrid strategy.

Figure 2 is plotted to demonstrate the dynamic properties of the *PHS* equilibrium, i.e., the phase portrait of the PHE (polymorphic hybrid equilibrium) with the hybrid strategy of Cindy (*C*_*2*_*, C*_*4*_) versus David (*D*_*2*_*, D*_*3*_) under following parameter values: *a* = *0.7*, *b* = 0.3, *c* = 0.1, *d* = 0.3, *k* = 0.5, and *m* = *0.6*. David and Cindy reach their decisions based on (*λ, μ*)=(0.498, 0.663), which is an equilibrium point as displayed as a red dot in the center of the red-cross in Figure 2. When *μ > 0.663,* Cindy prefers to choose *C*_*2*_ over *C*_*4*_, as indicated by the direction of portrait arrow (to left). When *μ < 0.663,* Cindy prefers to choose *C*_*4*_ over *C*_*2*_, as indicated by the direction of portrait (to right). When *μ = 0.663,* the *mixed strategy of C*_*2*_ and *C*_*4*_ is chosen. Similarly, David makes his strategic decisions based on the value of parameter *λ > 0.498, <0.498 or =0.498.*

### Influences of the cost, risk, and cooperation/communication factors on the policies

#### Influences of Cindy’s cost parameter (a) on the occurrences of the policies

**Figure 3:**
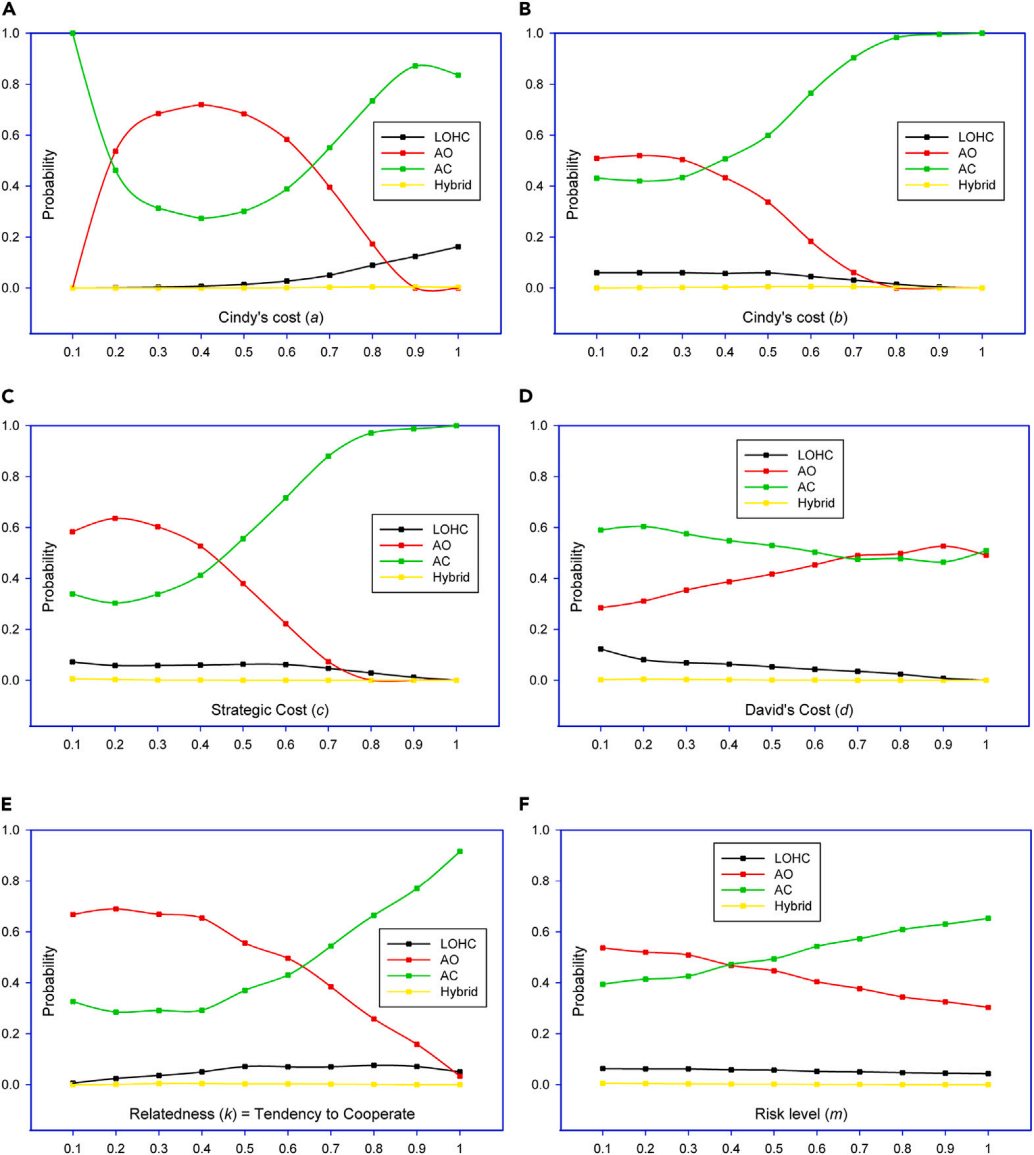
The probabilities of adopting the three policies estimated under different CDC parameter values The probabilities of adopting the three basic policies (LOHC, AO, and AC, respectively) estimated from 10^8^ simulations of the CDC game dynamics under different CDC parameter values: the fourth item “Hybrid” (i.e., PHE = polymorphic hybrid equilibrium) may be classified into one of the three previously listed basic policies, depending on complex interactions between Cindy and David; however, its probability and practical implications are negligible (*See* explanations below for specific Figures 3A–3F). (A) The probabilities of adopting three policies (LOHC, AO, AC) estimated from 10^8^ simulations of CDC game under different *cost* parameter (*a*) of Cindy. With the rise of Cindy’s cost (*a*) to underestimate the risk, the probability of adopting AO increases initially and reaches an asymptote at intermediate-risk level, and then declines steadily; that of adopting AC exhibits an opposite trend; and that of adopting LOHC policy is rather low but stable. The probability from adopting a policy generated by the hybrid strategy (i.e., PHE) is negligible. (B) The probabilities of adopting three policies (LOHC, AO, AC) estimated from 10^8^ simulations of CDC game under different *cost* parameter (*b*) of Cindy. With the rise of Cindy’s cost (*b*) to overestimate the risk, the probability of adopting AC increases gradually and reaches an asymptote; that of adopting AO exhibits an opposite trend; and that of adopting LOHC policy is rather low but stable. The probability from adopting a policy generated by the hybrid strategy (i.e., PHE) is negligible. (C) The probabilities of adopting three policies (LOHC, AO, AC) estimated from 10^8^ simulations of CDC game under different values of *strategic cost* (*c*): With the rise of strategic cost (*c*), the probability of adopting AC increases steadily and reaches an asymptote; that of adopting AO exhibits an opposite trend; and that of adopting LOHC policy is rather low but stable. The probability from adopting a policy generated by the hybrid (i.e.*,* PHE) strategy is negligible. (D) The probabilities of adopting three policies (LOHC, AO, AC) estimated from 10^8^ simulations of CDC game under different values of David’s *cost* (*d*): With the rise of David’s cost (*d*), the probability of adopting AO increases slowly and but declines when the cost is exceptionally high (>0.9); that of adopting AC exhibits an opposite trend; and that of adopting LOHC policy is low and decline slightly. The probability from adopting a policy generated by the hybrid strategy (i.e.*,* PHE) is negligible. (E) The probabilities of adopting three policies (LOHC, AO, AC) estimated from 10^8^ simulations of CDC game under different *relatedness* parameter (*k*): With the increase of *relatedness* (*k*) between Cindy and David (their collaboration tendency), the probability of adopting AO declines steadily; that of adopting AC increases gradually; and that of adopting LOHC is low but largely stable. The probability from adopting a policy generated by the hybrid strategy (i.e.*,* PHE) is negligible. (F) The probabilities of adopting three policies (LOHC, AO, AC) from 10^8^ simulations of CDC game under different risk level: With the rise of risk level (*m*), the probability of adopting AO declines steadily; that of adopting AC increases gradually; and that of adopting LOHC is low but stable. The probability from adopting a policy generated by the hybrid strategy (i.e., PHE) is negligible.

Figure 3A plotted the estimated probabilities of adopting three policies (LOHC, AO, AC) from *10*^*8*^ simulations of the CDC game under different cost parameter *a* (underestimating the risk) of Cindy. With the rise of Cindy’s cost (*a*) to underestimate the risk (*m*), the probability of adopting AO increases initially and reaches an asymptote under intermediate cost level (*a*≈0.3–0.5), and then decline steadily; that of adopting AC exhibited an opposite trend; and that of adopting LOHC policy is rather low but stable. The probability from adopting a policy generated by PHE (polymorphic hybrid equilibrium) is negligible. These probability trends appear natural. For example, when the cost (*a*) is relatively small (*a*<0.3), AO policy is favored (AC is disfavored), but when the cost is large (*a*>0.6), the trend is reversed. The cost parameter seems to have little influence on the likelihood of LOHC policy except when the cost is extremely high (*a*>0.7 approximately). The explanation for the negligible PHE probability is likely because of unduly complexity in implementing the PHE strategy. Indeed, we postulate that the small-probability associated with LOHC and PHE is because of their unduly complexities.

#### Influences of Cindy’s cost parameter (b) on the occurrences of the policies

Figure 3B plotted the estimated probabilities of adopting three policies (LOHC, AO, AC) from 10^8^ simulations of the CDC game under different cost parameter *b* (overestimating the risk) of Cindy. With the rise of Cindy’s cost to overestimate the risk (*b*), the probability of adopting AC increases gradually and reaches an asymptote; that of adopting AO exhibited an opposite trend; and that of adopting LOHC policy is rather low but stable. The probability from adopting a policy generated by PHE is negligible. These probability trends appear natural. For example, when the cost (*b*) of overestimation increases, AC policy is increasingly favored, and AO policy is increasingly disfavored.

#### Influences of strategic cost (c) on the occurrences of the policies

Figure 3C plotted the influence of strategic cost (*c*) on the likelihoods of the three policies (LOHC, AO, AC). With the rise of strategic cost (*c*), the probability of adopting AC increases steadily and reaches an asymptote; that of adopting AO exhibited the opposite trend; and that of adopting LOHC policy is rather low but stable. When the cost (*c*) > 0.6, the likelihood of adopting LOHC declines slightly, which may have to do with the possibly rising difficulty in effectively implementing the LOHC policy. The probability from adopting a policy (which could be any of the three policies, AC, AO, LOHC) generated by PHE (polymorphic hybrid equilibrium) is negligible, and little influenced by the strategic cost (*c*). These probability patterns again seem natural. For example, with the rise of strategic cost (*c*), the policy becomes more conservative and increasingly in favor for AC and against AO.

#### Influences of David’s cost (d) on the occurrences of the policies

Figure 3D illustrated the influence of David’s cost parameter (*d*) on the probabilities of three policies (LOHC, AO, AC) from 10^8^ simulations of the CDC game. With the rise of David’s cost (*d*), the probability of adopting AO increases slowly, but declines when the cost is exceptionally high (>0.9); that of adopting AC exhibited the opposite trend; and that of adopting LOHC policy is low and decline slightly. The probability from adopting a policy generated by PHE (polymorphic hybrid equilibrium) is negligible. When David’s cost is exceptionally high (*d* > 0.9), the policy is likely to reverse, which seems rather natural.

#### Influences of relatedness (k) on the occurrences of the policies

Figure 3E illustrated the influence of relatedness parameter (*k*) on the probabilities of adopting three policies (LOHC, AO, AC) from 10^8^ simulations of the CDC game. With the increase of relatedness (*k*) between Cindy and David (their collaboration tendency), the probability of adopting AO declines steadily; that of adopting AC increases gradually; and that of adopting LOHC is low but largely stable. The probability from adopting a policy generated by PHE (polymorphic hybrid equilibrium) is negligible. The finding seems natural again. For example, when the collaboration tendency increases, the AC policy is favored increasingly.

#### Influences of risk level (m) on the occurrences of the policies

Figure 3F illustrated the influence of pandemics risk (*m*) on the probabilities of adopting three policies (LOHC, AO, AC) from 10^8^ simulations of the CDC game. With the rise of risk level (*m*), the probability of adopting AO declines steadily; that of adopting AC increases gradually; and that of adopting LOHC is low and almost constant. The probability from adopting a policy generated by PHE (polymorphic hybrid equilibrium) is negligible. When the risk level *m* = 0.4, the probabilities of AC and AO crossover with each other, at which both policies are equally likely.

### Overall likelihoods (probabilities) of the three policies (LOHC, AO, AC)

**Figure 4:**
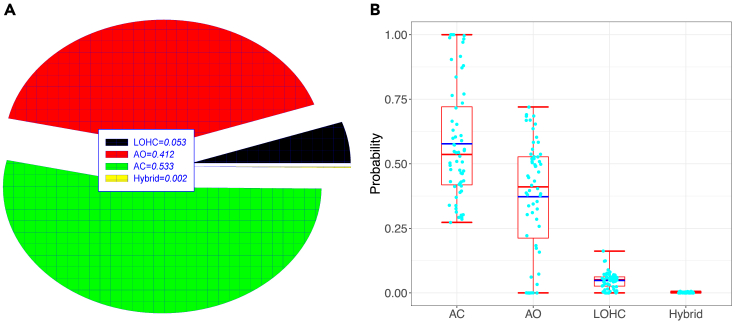
The probabilities of adopting the three policies estimated *across* all possible parameter settings The fourth item—hybrid (i.e., PHE)—can be classified into one of the three basic policies, depending on complex interactions between Cindy and David; nevertheless, its probability is negligible and so is its practical implications. Figure 4A (pie chart) shows the ‘partition’ of the probability space (‘pie’). Figure 4B shows standard box chart, with blue-colored line representing for the mean, red-colored line for the median, top box red-line for 75% points, bottom box red-line for 25% points, as well as the minimum (bottom red-line) and maximum (top red-line). The insights from the CDC game simulation are clear: (*i*) The universally accepted LOHC (low-risk open and high-risk closed) appears ideal but hardly implementable in practice, as evidenced by slightly larger than the probability of small event (p = 0.053). (*ii*) Highly complex polymorphic hybrid strategy (PHS) interactions seem promising, which theoretically can generate any of the three strategies (LOHC, AO, AC) depending on complex parameter setting (i.e., strategy interactions between Cindy and David), but the estimated probability is negligible (p = 0.002). (*iii*) The extensive simulations from the CDC game suggest that KISS (keep it simple strategically and persistently) should be large-probability events, as indicated by the rather high probabilities of AO (always open: p = 0.412) and AC (always closed: p = 0.533) policies. (*iv*) Finally, the HOLC (High-risk open & low-risk closed) policy, which is not displayed here, is obviously anti-science and unnatural, and it may be generated from strategies B & E (Table 1), but they do not have equilibriums, which implies evolutionarily unstable and is unlikely to sustain in practice.

In the previous section, we discussed the influences of single factor (CDC parameter) on the policy-making. In this section, our focus is on the overall probabilities of the three policies. In Figure 4, we plotted the overall probabilities of adopting three basic policies (LOHC, AO, AC), respectively, across all simulated parameter settings (i.e., averaged from the values across all parameters or Figures 3A–3F). The fourth item—PHE—can be classified into one of the three basic policies, depending on complex interactions between Cindy and David. However, to classify each PHE into one of the three policies, additional ‘real-time’ (i.e.*,* the threshold value of parameter *λ* or *μ* determines the choice of mixed strategy) interaction information is necessary, which requires additional extensive simulations. Because the total (combined) probability (p = 0.002) of the three policies from PHE is negligible, the lack of further classifications should only have negligible biases on the probabilities of the three policies drawn in Figure 4.

From Figure 4, findings on the overall likelihoods (probabilities) of the three policies include: (1) The near universally adopted LOHC, i.e., a middle-ground of AC and AO policies, intuitively should be preferred, but can be too complex to enforce practically, as suggested by the only slightly larger than the probability of small event (p = 0.053). (2) Highly complex polymorphic hybrid strategy (*PHS*) interactions seem promising, which theoretically can generate any of the three strategies (LOHC, AO, AC) depending on complex parameter setting (strategy interactions between Cindy and David), but the estimated probability is negligible (p = 0.002). (*i*3) The extensive simulations (*10*^*8*^ times) of the CDC game suggest that KISS (keep it simple strategically) should be large-probability events, as indicated by the rather high probabilities of AO (always open: p = 0.412) and AC (always closed: p = 0.533) policies. (4) Finally, the HOLC (high-risk open and low-risk closed) policy, which is not displayed in Figure 4, is obviously anti-science and unnatural. Although LOHC may be generated from strategies B & E (Table 1), both strategies B & E do not have equilibriums, which means that they are evolutionarily unstable and unlikely to sustain in practice.

### Payoffs of the CDC game players

**Figure 5:**
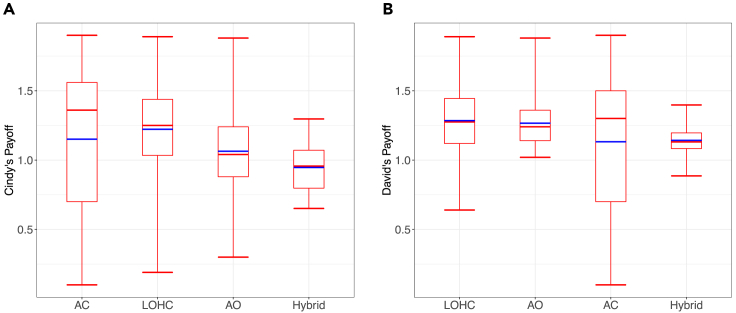
Cindy’s and David’s payoffs under different policies are illustrated as standard box charts respectively Notably, the order of payoffs is different for Cindy (the left chart) and David (the right). With Cindy’s payoff, the descending order (from left to right) is *AC>LOHC>AO>Hybrid*, and with David’s payoff, the descending order is *LOHC>AO>AC>Hybrid*. The orders are statistically significant per Wilcoxon tests (p-value < 0.05). Note the scatter points are omitted in the box charts to avoid overwhelmingly large number of points, which could lead to overly big file size of the graph.

In previous sections, our discussions are focused on the simulation of the influences of various CDC game parameters (that represent for various costs/benefits, risk and cooperation/communication factors) on the strategies (policies). In this section, our focus is the payoffs of CDC game. Figure 5 plotted the payoffs of Cindy and David under different policies, respectively. Notably, the order of payoffs is different for Cindy (Figure 5A) and David (Figure 5B). With Cindy’s payoff, the descending order (from left to right) is AC>LOHC>AO>Hybrid, and with David’s payoff, the descending order is LOHC>AO>AC > Hybrid. The orders are made based on statistically significant differences based on Wilcoxon tests (p-value < 0.05). The opposite payoff-orders suggest that Cindy prefers tighter AC policy, whereas David prefers more relax AO policy. This difference may be due to the reality that David should be more concerned with the health versus wealth tradeoff or dilemma.

## Conclusions and discussion

### Conclusions

The main insights from the CDC game can be summarized as follows:

(1) Among the 16 (4x4) equivalently reciprocal strategy interactions of the CDC game, approximately ½ are unnatural or irrational, which allows us to capture not only appropriate strategies but also those unnatural (or irrational) strategies that might be generated by faulty judgment(s) and/or miscommunications in policy-making system. An advantage of our CDC game approach is the completeness of the 16-elements strategy set in a two-player game.
(2) The CDC game has six types of equilibriums, which are evolutionarily stable and can be sustainable in practice. Some of the equilibriums are Nash equilibrium, which means that no game player would regret for his or her strategy. Some are pooling equilibrium, which can be roughly considered as “dictatorship-style”; in the case of CDC game, the pooling equilibrium implies that David would ignore Cindy’s advice. Some are separating equilibrium, which can be roughly considered as the opposite to pooling equilibrium, i.e., Cindy’s advice does make differences. The so-termed polymorphic hybrid equilibrium (PHE) means that sometimes the equilibrium is pooling and sometimes separating.
(3) There are four basic anti-pandemic policies: LOHC, HCLO, AO, and AC. They can be mapped to the output of CDC game system, i.e.*,* the ultimate decision made by David in the CDC game. The LOHC is arguably the most rational policy and has been adopted by the majority of countries (regions) in the world. The diametrically opposite HOLC is obviously the least rational, and it is unlikely being adopted unless fatal mistake occurs. Although the HOLC can be mapped to strategy interactions B & E, both do not have any equilibriums, mirroring the reality that the policy is unlikely to sustain in practice. In contrast, the LOHC policy can be mapped to strategy interactions, *A*, *F*, and *PHS* (polymorphic hybrid strategy), with *A* being Nash equilibrium, *F* being ESS (evolutionarily stable strategy), and *PHS* being hybrid (sometimes pooling and sometimes separating). The simulation (of 100-millions times) with the CDC game revealed that the probability of LOHC is small-probability event (*P* = 0.053), whereas the probability of HOLC is zero. The small-probability event characteristic of LOHC appears to be counter-intuitive, and we postulate that practical implementation of LOHC can be too complex to be feasible, especially for the COVD-19 pandemic that has been recurring one wave after another. The multi-wave and extreme contagiousness of COVID make failure in one round of reopening sufficiently ruin the whole efforts, i.e.*,* cascading failures. The small probability of LOHC seems to mirror a reality that, although most countries have adopted it, arguably and at least on the surface, obviously most countries have not achieved their expectations.
(4) The AO policy can be mapped to the equilibriums of mixed strategy of *J* & *K*, or *PHS.* The mixed strategy *J* & *K* are pooling equilibrium, which means that the CDC chief scientist’s advice makes no difference or is ignored by the ultimate decision-maker, David. The hybrid strategy *PHS* may also generate AO policy, and in this case, sometimes the chief’s advice matters and sometimes does not. The simulation of 100-million times with the CDC game revealed that the probability of AO policy is *P* = 0.412, and that of *PHS* is negligible (*P* = 0.002). Therefore, virtually all AO policies are generated from the mixed-strategy of *J* & *K*, which is from David’s “dictatorship-style” decision (a characteristic of pooling equilibrium).
(5) The AC policy can be mapped to the equilibriums of mixed strategy *I* & *L*, or *PHS.* Simulations revealed that the probability of the AO policy is *P* = 0.533, and that of *PHS* is negligible (*P* = 0.002). Similar to the previous AO policy, the equilibriums of *I* & *L* are pooling, and therefore, Cindy’s advice usually does not matter in the making of AC policy.
(6) Simulations of 100 million times suggested that the order of probabilities of three policies is AC>AO > LOHC, while HOLC = 0. Of interest, in terms of the payoff, Cindy and David have different orders. For Cindy, her payoff order is AC>LOHC>AO; for David, his payoff order is LOHC>AO>AC. Note that these three orders are of different nature. The first order is the likelihood of policy order, which is the product of the CDC game, or the outcome of Cindy and David strategic interactions. The second order is Cindy’s preference (payoff) order, which should largely depend on her professional expertise; her profession as CDC chief obviously should have pushed her to prefer AC, i.e., her payoff (such as reputation or satisfaction from saving lives) is maximized. The third order is David’s preference (payoff) order, which should largely depend on his duty as the president who needs to balance health versus wealth. For president, AC is likely his least favorite, whereas LOHC is ideally his top choice although it is often too complex to implement. Therefore, although both Cindy and David seem to have different preferred strategies, their interactions produce a single set of “meta-strategies”, i.e., the policy set of LOHC, AO, and AC.
(7) More or less, most countries (regions) in the world seemed to have adopted the LOHC policy, whereas a handful of countries have adopted *laissez-faire* strategy such as passive herd immunity that may be considered as AO policy. The dynamic zero-out policy adopted by several countries (notably China and New Zealand) seemed to be equivalent to AC in terms of the effects, and to “precision” LOHC operationally in terms of the implementation.

In conclusion, through rigorous analytic and extensive simulation analyses of the CDC game, we obtained reasonable interpretations for the three fundamental public-health policies against the COVID-19 pandemic, i.e., AO, AC and their middle ground LOHC, precluding the obviously irrational or anti-intelligent HOLC policy. The policy of AO is likely to be wealth-optimal, while the policy of AC is likely to be health-optimal. The LOHC may achieve some level of balance between health and wealth, given it is a middle ground of both AC and AO. The HOLC generally favors neither health nor wealth, what we called anti-intelligent and is not sustainable (corresponding to no equilibriums in the CDC game). In real world, most countries (regions) have adopted the LOHC policy, which seems to balance the health versus wealth dilemma and appears to be an optimal policy choice. However, simulations suggest that the policy may be too complex to implement successfully given its probability is only 0.053, a typical small probability event. In contrast, the two ends of the policy spectrum, AO and AC exhibited large probabilities (close to 0.41–0.53). A take-home message seems to be KISS (keep it simple strategically and persistently), and the large probabilities of AC and AO are likely due to their simplicity.

### Discussion

Humans are believed to possess exceptional evolutionary capacity for decision-making, which should have contributed to the dominance of the humans on planet earth (Samson 2020).^33^ Although on macroscopic scale, humans have been enormously successful from selecting the right food and shelter, to devising complex economic strategies and effective public health policies (Samson 2020)^33^, we have occasionally made expensive and painful mistakes, locally, regionally and globally, including the strategies and policies for fighting the COVID-19 pandemic. The challenge seems particularly serious in the era of disinformation (e.g., Bergstrom & West 2020).^34^ Given the nature of the complexity and challenges, we formulate the decision-making problem for anti-COVID-pandemic as the CDC game, which is an extension to the classic SPS game. The latter achieved wide success in studying the reliability (honesty) of animal communications in evolutionary biology. The SPS game, especially its extensions (Huttegger & Zollman 2010, Whitmeyer 2020, Ma & Zhang 2022) are particularly powerful in modeling the processes that are driven by 3C forces (cooperation, competition and communication).^9^^,^^13^^,^^14^ Since we are only interested in strategic decision-making, it is natural for us to focus on the stable equilibriums. Tactical or operational level analyses are not considered in this article.

There are 16 (4x4) possible strategy combinations (interactions) with the CDC game given it is an asymmetric four-by-four strategic-form game. Also given the completeness of the strategy interactions (4x4), the strategy interactions are in effects *reciprocal*. That is, there is likely an irrational (or unnatural) counterpart for each rational (or natural) strategy interaction. Given the equivalently reciprocal nature of the CDC game, it is expected that approximately 1/2 of the strategy interactions can be unnatural. Furthermore, there are *six* types of *equilibriums* (Table 2) among the 16 possible strategic interactions (Tables 1 and 2). The *six equilibriums* can be classified as separating (signaling), pooling, and polymorphic hybrid equilibriums, and they may possess different behavioral and payoff properties (*see* Table 3, Box 1 and 2). These equilibriums and stabilities can be significantly different, with different theoretical ramifications (e.g., Maynard-Smith & Harper 2003, Huttegger & Zollman 2010, Bergstrom & Lachmann 1997, 1998, Biernaskie et al. 2018, Madgwick & Wolf 2020, Whitmeyer 2020).^6^^,^^7^^,^^8^^,^^9^^,^^14^^,^^35^^,^^36^ Practically, these six equilibriums can be mapped to three basic anti-pandemic policies.

Among the four fundamental anti-pandemic policies, the LOHC (low-risk open and high-risk closed) is essentially a middle ground of the two extreme policies: AC (always closed) and AO (always open). The LOHC policy appears to be the most rational and apparently most effective, and it has obviously been adopted by most countries (regions) of the world for fighting against the COVID19 pandemic. Herd immunity or co-existence policies can be considered as AO policies. The strict AC policy, although medically optimum but economically risky, seems to be the least popular. In summary, co-existence with virus versus zero-out virus appears to mirror the AO versus AC policies, while the LOHC policy seems to be the middle ground of both AO and AC. The unnatural HOLC (high-open and low-closed) is irrational, and has been adopted by nobody in the world. Strategy interactions B & E can be mapped to the HOLC policy, but they do not have any equilibriums, which is expected and simply mirrors the unsustainable nature of irrational HOLC policy.

Approximately half of the 16 (4x4) strategies can produce equilibriums, and the equilibriums can be mapped to the three fundamental policies, assuming those equilibriums are sustainable. For the approximately half of the strategy interactions that do not have corresponding equilibriums, their strategies are unsustainable in practice and may mirror some of the unnatural and idiosyncratic behaviors in anti-pandemic policy-making. The explanation for the approximately ½ natural versus unnatural strategies or with/without corresponding equilibriums should be to do with the reciprocal nature of the strategy set (options) as explained previously. Although it is difficult to interpret all of the unnaturalness or the idiosyncrasy in half of the strategy set, there are some answers from the recent studies in behavior economics (Samson 2014, 2020).^33^^,^^37^ Studies have revealed that humans are not always self-interested, benefits maximizing, and costs minimizing with stable preferences. Human minds are not always rational and may only possess *bounded rationality*, which suggests that human rationality is limited by brain’s information processing capability, insufficient knowledge feedback, and time constraint (Ma 2015a, 2015b, Samson 2014, 2020), not to mention complex politics in the case of the CDC game.^33^^,^^37^^,^^38^^,^^39^ The fact that approximately ½ of the possible strategy options seem to demonstrate the rather high likelihood to devise an unsustainable (faulty) policy. In other words, behaving rationally should not be considered as granted for the CDC-president interactions.

In contrast, approximately half of the strategy interactions with possible equilibriums can be sustainable, and mapped to one of the three basic policies (AO, AC, & LOHC). However, equilibriums, stability, and rationality do not necessarily correspond to scientifically optimal, and may not even necessarily correspond to scientific correctness, since the CDC output (policies) may also be influenced by politics or other socioeconomic factors. This is determined by the aim of this study—identifying the potential cognitive gaps and possibly traps by emulating the reality of anti-pandemic policies with the CDC game model.

The AO policy is essentially the *laissez-faire* strategy (e.g., passive herd immunity), which was initially adopted by a handful of countries including Sweden and partially by UK. The strictly AC seems unrealistic intuitively, but theoretically it does corresponds to two strategies with stable equilibriums. In practice, the dynamic zero-out (infections) policy adopted in China and New Zealand can be considered as either precision version of LOHC in terms of implementation, or an equivalent AC policy in terms of the effects. The dynamic zero-out policy does have a precondition, namely, it must be feasible to control the infection level below the threshold of *Allee effect*, and therefore, the infections level should be relatively lower in general to further push it below the threshold (of “local extinction”).

The Allee effect is named after animal ecologist W. C Allee (Allee & Bowen 1932)^17^, and it is a theoretic threshold of population growth (such as the infections of COVID-19), above which population growth may accelerate and below which population growth may decelerate and may go extinct ultimately (Kramer et al. 2017).^40^ Its implications to epidemiology have been discussed in recent literature; for example, Friedman et al. (2012) studied the relationship between the Allee effect of disease pathogens and the Allee effect of hosts (e.g., humans) to answer a question of fundamental importance in epidemiology: Can a small number of infected individual hosts with a fatal disease drive the host population to go extinct (assuming a healthy stable host population at the disease-free equilibrium is subject to the Allee effect)?^41^ Ma (2020) estimated the population aggregation critical density, the threshold for aggregated (clustered) infections of COVID-19 to occur.^42^ Besides Allee effects, two other theories, i.e., metapopulation dynamics and tipping-point theories, are particularly important for supporting the dynamic zero-out policy. The metapopulation theory maintains that extinctions of local population are common events, and tipping point theory suggests that, at some critical points, population growth may transit to either outbreak or die-off from the tipping point (threshold) (Citron et al. 2021).^16^ There are techniques for detecting tipping points by identifying some early warning signals (EWS), which can help to implement dynamic zero-out policy. These theories suggest that there are thresholds (tipping points) at which local pathogen (infections) may go extinct, depending on the dynamics of host-pathogen system and disease control measures. Therefore, public health policies designed to drive the infections (such as SARS-COV-2) to go extinct *locally* (below the threshold of Allee effect of the pathogen), whereas keeping the host population absolutely safe from the risk associated with the Allee effect of the hosts (this is, of course, not an issue for humans at all), should be feasible. In other words, the dynamic zero-out policy, which is equivalent to driving *local* infections to extinctions, is theoretically feasible when implemented properly. The practices of dynamic zero-out policy during much of the COVID-19 pandemic period by a handful of countries, notably China and New Zealand, have demonstrated its feasibility and success.

### Limitations of the study

There exist a few limitations with our study, as rightly pointed out by the anonymous reviewers of our article. One possible limitation is that our analysis (the analytic results) of the CDC game model was based on infinite population sizes of the game players, although the simulations were of limited population sizes, as rightly pointed out by the anonymous reviewers. Existing studies have revealed that the finite population sizes may influence the equilibriums of the SPS game (e.g., Catteeuw 2013, 2014, Tanimoto 2015, 2019, 2021).^25^^,^^26^^,^^43^^,^^44^^,^^45^ Another limitation of our study is that the CDC model does not have an explicit and direct punishment/reward mechanism to either Cindy or David, unlike some existing studies (e.g., Miyaji et al. 2013, Rand et al. 2013, Zisis et al. 2016, Tanimoto 2015, 2019, 2021, Kabir et al. 2021, Hossian & Tanimoto 2022, Zhou et al. 2022) in which punishment/reward mechanisms are implemented to represent various social behaviors and cultures such as pro-social behavior, generosity, fairness, and good citizenship.^25^^,^^26^^,^^45^^,^^46^^,^^47^^,^^48^^,^^49^^,^^50^^,^^51^ In addition, our CDC game model ignored many practically significant factors such as vaccinations (Piraveenan et al. 2021), heterogeneity of populations (such as different occupations) (Hancean et al. 2022), and the evolution (variations) of pathogens (SARS-COV-2), regional and global cooperation (Czypionka et al. 2021, Priesemann et al. 2021, and Valdez 2022).^52^^,^^53^^,^^54^^,^^55^^,^^56^ Although we do believe that these additional factors are critical for the decision-making of anti-COVID pandemic, the game theoretical analysis should focus on strategy level, and those additional factors can be investigated at tactical and operational levels. In this aspect, the so-termed three-layer survivability analysis (Ma 2008, Ma & Krings 2011), in which traditional engineering reliability theory and survivability theory for survivable network systems were unified in a hierarchical framework consisting of the strategic, tactical and operational levels, may be applied to develop a decision-support system with strong operability for the anti-pandemic decision-making.^12^^,^^57^ The three-layer survivability analysis framework consists of survival analysis (reliability analysis) at tactical level, dynamic hybrid fault models with the so-termed “Byzantine Generals playing evolutionary game”, and the optimization supported with hedging principle and evolutionary computing.^12^^,^^57^ The embedding of the CDC game modeling into the three-layer survivability analysis framework should be able to provide a blueprint for building a decision-support system for managing pandemics, including possible future public health crises.

In addition, what epidemiology studies are typical complex dynamic systems, from emergence, epidemic, through pandemic to endemic stage. Prediction *per se* is a rather challenging task, especially for dynamic systems such as COVID-19 pandemic (Vytla et al. 2021, Ma 2020, 2021, 2022).^42^^,^^58^^,^^59^^,^^60^ According to Vytla et al. (2021) survey, the prediction of the COVID-19 pandemic can be characterized as “the kryptonite of modern AI” and “many predictions by AI and machine learning were neither accurate nor reliable.”^58^ Properly predicting the stage transitions and associated EWS (early warning signals) for the tipping points of epidemics/pandemic is another critical mission of decision-making body such as CDC, which is necessary for timely and smooth adaptations/transitions of anti-pandemic strategies/policies. To further illustrate the point, we end the article with recording following *fictitious* transcript of a phone conversation between Cindy and David.

We emphasize that the fictional scenario below related to the COVID pandemic is in no reference to any actual conversation between any real persons. It should also be reiterated that the dialogue only represents an idealized scenario out of 16 possible interactions of the CDC game, actually a possibly dynamic mixture of the interactions based on specific stages of the pandemic. Readers are free to infer their own thoughts from the conversation. However, the authors take no standings on the conversations and their intentions and interests are limited to the pure mathematical and simulation analyses of the CDC game model *per se*. If there is any implicit expression of personal opinions from the authors, that is the call for mutual understanding and reconciliation between the AO (always open) and AC (always closed) supporters. In the end, given that the virus-host interaction is a dynamic system, which is deeply interwoven with socio-economic systems, our strategies to intervene the interaction systems in favor for humans, whether it is to extinguish, contain, or accommodate the virus, should be dynamically adaptive with the evolution of virus, the development of economy and impacts from social and political environments. In other words, the optimal policies should be stage (emergence, outbreak, pandemic, endemic) dependent.

Assume the following contrived scenario: It’s the year-end of 2022. President is planning for his New Year’s speech, and he picks up the phone and calls Cindy.

*David*: Hey, how are you doing Cindy? This is David.

*Cindy*: Mr. President, I am just fine, [coughing …] excuse me, Mr. President.

*David*: Cindy, are you OK? Are you sure?

*Cindy*: Excuse me, David, it’s been over a week, but you know, it should have been over by now. Please do not worry. I am fine, Mr. President.

*David*: I was thinking about my New Year’s speech and possible questions from reporters about the pandemic, you know.

*Cindy*: Yes, Mr. President. I think it will be over next year, and this should be the last New Year’s briefing they are still interested in this topic.

*David*: Right, Cindy. It has been three whole years. I still recall our first meeting three years ago. You were almost shouting, at me, “Mr. President, it’s time to shut down … and enact the quarantine immediately.” You were right and saved all of us from the first wave!

*Cindy*: Thank you! David, I have always been appreciative of your trust! You know, it was not only feasible, but also the most cost-effective strategy during the initial outbreak.

*David*: That’s right! But not long later we had to enter the cycles of lockdown-reopen … one round after another. Frankly, I had somewhat lost patience occasionally, certainly not with you, Cindy, but with the cascading ups and downs, locally, nationally, and continently. You were telling me your LOHC repeatedly … economy began to suffer … people losing patience … In the end, we saved millions of lives while avoiding stagnation of the economy … that’s a fact! I thank you for your persistence and service to our country.

*Cindy*: David, it was your wise calls! I only did what I was supposed to do.

*David*: You are too generous! It was your thorough, timely, and convincing analysis that persuaded me! I just wonder if we could have lifted all the controls earlier.

*Cindy*: I wish we could! But David, you know, it’s all about timing! Omicron is a frenemy variant, and our immune systems can usually beat it just like overcoming the flu.

*David*: What about long-COVID and the possibility of a new vicious variant? Which seem to be a current focus of public concerns.

*Cindy*: Mr. President, I understand. Nobody has a crystal ball, but I believe the chance of a vicious variant is rather small. In case I am wrong, we may have to restart, but I am confident that we will do it even better! As to long-COVID, it is beyond my expertise, as you know, Mr. President. My suggestion would be to increase funding for research.

*David*: Thank you! Cindy, I fully agree. But I am a little bit concerned if I can persuade the public for funding. You know, given that we do not yet have an effective drug to cure HIV, not to mention COVID, after so many years of extensive funding for research.

*Cindy*: I understand those concerns, Mr. President! Funding for research, especially for basic research, is not unlike venture capital investments, if not lottery. Your gains are huge when you hit.

*David:* Finally, Cindy, I hmm, I’d like to know your opinion about a somewhat awkward matter, but it’s absolutely nothing personal, and I respect and admire experts, trust me. But have you heard a research paper reporting only 4% fever rate with COVID?^61^ Interestingly, the study was done in the March and the paper was published in the June [of 2022].

*Cindy*: [Coughing and laughing …] Mhm. Mr. President, I happened to hear it from my assistant who was rather reserved and puzzled with the findings. He is now rather upset after he has experienced the infection himself. Seriously, I think it might be a benign lie, rather than payola scheme, as debated between the fans and doubters. Unfortunately, the paper is gone now!^61^

*David*: Could it be hacked?^61^

*Cindy*: Uh-oh, I hope so!

*David*: Well, let’s put it behind and celebrate the New Year! By the way, should we now focus on speeding up the research and development of cancer vaccines?

*Cindy*: Yes, sure! Mr. President. You are always being so kind! I wish you a very Happy New Year!

## Ethic approval

N/A because the study does not involve any wet-lab experiments or survey on human or animal subjects, and all analyzed datasets are already available in public domain, as mentioned above.

## STAR★Methods

### Key resources table

**Table:** 

REAGENT or RESOURCE	SOURCE	IDENTIFIER
**Software and algorithms**		

Python Code for Simulation of CDC game	This paper	Online Supplemental Information: Simulation-Equilibriums.ipynb
Matlab Code for Generating Policies	This paper	Online Supplemental Information: Policy.m
Python Version 2.7.5	Python Software Foundation	https://www.python.org
Matlab Version 2018a	MathWorks®	https://www.mathworks.com

### Resource availability

#### Lead contact

Further information and reasonable requests for resources and reagents should be directed to and will be fulfilled by the lead contact, Sam Ma (ma@vandals.uidaho.edu).

#### Materials availability

This study did not generate new unique reagents.

### Method details

Ongoing debates on anti-COVID19 policies have been focused on coexistence *vs.* zero-out strategies, which can be simplified as “always open (AO)” *vs*. “always closed (AC).” We postulate that, a middle ground between the two extremes, dubbed LOHC (low-risk open and high-risk closed), is likely more favorable, precluding obviously irrational HOLC (high-open-low-closed). From a meta-strategy perspective, these four policies cover the full spectrum of anti-pandemic policies. We argue that, among numerous factors influencing strategic policy-making, the competence of advisory body such as CDC chief-scientist (*say*, Cindy) and politics in decision-making body such as president (David), and their cooperation/communication can be critical. Here we investigate anti-pandemic policy-making by harnessing the power of evolutionary game theory in modeling competition/cooperation/communication (three critical processes underlying biological and social evolutions). Specifically, we apply the Sir Philip Sydney (SPS) game, a 4x4 signaler-responder evolutionary game with 16 strategic interactions, which was devised to investigate the reliability of animal communication that can modulate competition and cooperation, to capture rich idiosyncrasies surrounding today’s anti-pandemic policies. By emulating the reality of anti-pandemic policies today, the study aims to identify possible cognitive gaps and traps. The extended SPS, dubbed CDC (Cindy and David’s Conversations) game, offers a powerful cognitive model for investigating the coexistence/zero-out dichotomy and possible alternatives.

The formulation of the CDC (Cindy and David’s Conversations) problem as an extended SPS (Sir Philip Sydney) game is formally exposed with the supports of Table 1, Figure 1, and Box 1. In particular, Box 1 below further exposed the three key elements (Who, What and How) to formulate the CDC game, the transformation from the SPS through to the CDC game, as well as the interpretations of the CDC parameters.

#### The differential equations of the CDC game model

We adopt the extended SPS game model formulated by Huttegger & Zollman (2010)^9^ to describe the CDC game. The two-population replicator dynamics for Cindy and David, based on Huttegger & Zollman (2010)^9^, can be written as follows:

Let *x*_*i*_ be the relative frequency of Cindy’s strategy type *i,* and *y*_*j*_ be the relative frequency of David’s strategy type *j*, where *i, j*=1, 2, 3, 4, the replicator dynamics is written as follows:

(Equation 1) $${\overset{\cdot }{x}}_{i}=x_{i}\left[\pi_{i}\left(\vec{y}\right)-\pi \left(\vec{x},\vec{y}\right)\right]$$

(Equation 2) $$\overset{\cdot }{y_{j}}=y_{j}\left[\pi_{j}\left(\vec{x}\right)-\pi \left(\vec{y},\vec{x}\right)\right]$$

where $\vec{x}$ =(*x*_1_, *x*_2_, *x*_3_, *x*_4_) and $\vec{y}$ =(*y*_1_, *y*_2_, *y*_3_, *y*_4_), are the strategy sets of Cindy and David respectively; $\pi_{i}(\vec{y)}$ is the payoff of *i* strategy against $\vec{y}$ and $\pi \left(\vec{x},\vec{y}\right)$ is the average payoff in Cindy’s population; $\pi_{j}(\vec{x)}$ is the payoff of *j* strategy against $\vec{x}$ and $\pi \left(\vec{y},\vec{x}\right)$ is the average payoff in David’s population.

### Quantification and statistical analysis

Wilcoxon test with threshold *P*-value=0.05 is used to determine statistical significance.

## Data Availability

• This study did not analyze any existing data and only generated the data of the simulated parameter sets for the CDC game equilibriums. The parameters are supplied in the online supplemental information (Tables S1, S2, S3 and S4). • The Python and Matlab codes (Data S1) are supplied in file “Data S1.PDF”, titled “The Readme file and Two Lists of the Source Code”. • No additional information is available.

## Supplemental information

Table S1. The list of equilibriums associated with the LOHC Policy (Figures 3, 4, and 5)

Table S2. The list of equilibriums associated with the AC (Always Closed) Policy (Figures 3, 4, and 5)

Table S3. The list of equilibriums associated with the AO (Always Open) Policy (Figures 3, 4, and 5)

Table S4. The list of polymorphic hybrid equilibriums (PHE) (Figures 3, 4, and 5)

Data S1. The Readme file and Two Lists of the Source Code (STAR Methods)
