# Supplementary material for: CDC (Cindy and David’s Conversations) game: Advising President to survive pandemic
Source: iScience. 2023 Jun 9;26(7):107079. doi: 10.1016/j.isci.2023.107079 (PMC10250248; doi:10.1016/j.isci.2023.107079)
Supplement: Data S1. The Readme file and Two Lists of the Source Code (STAR Methods) [file mmc5.pdf]

Data S1/Methods S1: The Readme file and Two Lists of the Source Code (Star★Method)

Ma & Yang (2022) CDC (Cindy and David's Conversations) Game: Advising President to Survive Pandemic."

(1) Generate_Parameters.ipynb
Python code for simulating the CDC game, generating the equilibrium points and computing the corresponding payoffs of the players.

(2) Policy.m
MATLAB script that classifies the equilibriums points from the previous Generate_Parameters.ipynb program into three types of anti-pandemic policies (LOHC, AC, AO & Hybrid).

Generate_Parameters.ipynb

Python source code starting from the next page (10 pages in total):

```
{
 "cells": [
  {
   "cell_type": "markdown",
   "metadata": {},
   "source": [
    "## generate the simulation data of equilibrium points"
   ]
  },
  {
   "cell_type": "code",
   "execution_count": null,
   "metadata": {},
   "outputs": [],
   "source": [
    "import numpy as np\n",
    "import pandas as pd\n",
    "from pandas import DataFrame\n",
    "def generate_parameters_A():         \n",
    "    roundNum = 3\n",
    "\n",
    "#     print(\"a b c d k m\")\n",
    "    result
=pd.DataFrame(columns=('a','b','c','d','k','m','Cindy_Payoff','David_Payoff'))  \n",
    "    row = 0\n",
    "    for a in np.arange(0.1,1,0.1) : \n",
    "        for b in np.arange(0.1,1,0.1) : \n",
    "            for c in np.arange(0.1,1,0.1) : \n",
    "                for d in np.arange(0.1,1,0.1) : \n",
    "                    for k in np.arange(0.1,1,0.1): \n",
    "                        for m in np.arange(0.1,1,0.1):\n",
    "                            a=round(a,roundNum)\n",
    "                            b=round(b,roundNum)\n",
```

```
    "                             c=round(c,roundNum)\n",
    "                             d=round(d,roundNum)\n",
    "                             k=round(k,roundNum)\n",
    "                             m=round(m,roundNum)\n",
    "                             if  (a>=k*d-c and k*d-c>=b and a>=d/k and d/k>=b):\n",
    "                                 Cindy_Payoff=1-b-c+k+m*(b+c-d*k)\n",
    "                                 David_Payoff=1+k*(1-b-c)+m*(k*b+k*c-d)\n",
    "                                 \n",
    "                                 result.loc[row] =
[a,b,c,d,k,m,Cindy_Payoff,David_Payoff] \n",
    "                                 row += 1                        \n",
    "     result.to_excel('A_data.xlsx',index=False)     \n",
    "generate_parameters_A() "
   ]
  },
  {
   "cell_type": "code",
   "execution_count": 2,
   "metadata": {},
   "outputs": [],
   "source": [
    "import numpy as np\n",
    "import pandas as pd\n",
    "from pandas import DataFrame\n",
    "def generate_parameters_F():\n",
    "     roundNum = 3\n",
    "     #     print(\"a b c d k m\")\n",
    "     result
=pd.DataFrame(columns=('a','b','c','d','k','m','Cindy_Payoff','David_Payoff'))  \n",
    "     row = 0\n",
    "     for a in np.arange(0.1,1,0.1) : \n",
    "         for b in np.arange(0.1,1,0.1) : \n",
    "             for c in np.arange(0.1,1,0.1) : \n",
```

```
    "                for d in np.arange(0.1,1,0.1) : \n",
    "                    for k in np.arange(0.1,1,0.1): \n",
    "                        for m in np.arange(0.1,1,0.1):\n",
    "                            a=round(a,roundNum)\n",
    "                            b=round(b,roundNum)\n",
    "                            c=round(c,roundNum)\n",
    "                            d=round(d,roundNum)\n",
    "                            k=round(k,roundNum)\n",
    "                            m=round(m,roundNum)\n",
    "                            if  (a>=c+k*d and c+k*d>=b and a>=d/k and d/k>=b) :\n",
    "                                Cindy_Payoff=1-b+k+m*(b-c-d*k)\n",
    "                                David_Payoff=1-b*k+k+m*(b*k-c*k-d)\n",
    "                                result.loc[row] =
[a,b,c,d,k,m,Cindy_Payoff,David_Payoff] \n",
    "                                row += 1                        \n",
    "    result.to_excel('F_data.xlsx',index=False)    \n",
    "generate_parameters_F()"
   ]
  },
  {
   "cell_type": "code",
   "execution_count": 3,
   "metadata": {},
   "outputs": [],
   "source": [
    "import numpy as np\n",
    "import pandas as pd\n",
    "from pandas import DataFrame\n",
    "def generate_parameters_L():\n",
    "    roundNum = 3\n",
    "    result
=pd.DataFrame(columns=('a','b','c','d','k','m','Cindy_Payoff','David_Payoff'))  \n",
    "    row = 0\n",
```

```
    "    for c in np.arange(0.1,1,0.1) : \n",
    "        for a in np.arange(0.1,1,0.1) : \n",
    "            for b in np.arange(0.1,1,0.1) : \n",
    "                for d in np.arange(0.1,1,0.1) : \n",
    "                    for k in np.arange(0.1,1,0.1) : \n",
    "                        for m in np.arange(0.1,1,0.1): \n",
    "                            c=round(c,roundNum)\n",
    "                            a=round(a,roundNum)\n",
    "                            b=round(b,roundNum)\n",
    "                            d=round(d,roundNum)\n",
    "                            k=round(k,roundNum)\n",
    "                            m=round(m,roundNum)\n",
    "                            if d<k*(m*a+(1-m)*b) :\n",
    "                                Cindy_Payoff=1+k*(1-d)\n",
    "                                David_Payoff=1-d+k\n",
    "                                result.loc[row] =
[a,b,c,d,k,m,Cindy_Payoff,David_Payoff] \n",
    "                                row += 1                       \n",
    "    result.to_excel('L_data.xlsx',index=False)                            \n",
    "generate_parameters_L()"
   ]
  },
  {
   "cell_type": "code",
   "execution_count": 4,
   "metadata": {},
   "outputs": [
    {
     "name": "stderr",
     "output_type": "stream",
     "text": [
      "E:\\Program Files\\Install\\Python\\lib\\site-packages\\ipykernel_launcher.py:22:
RuntimeWarning: divide by zero encountered in double_scalars\n"
```

```
      ]
     }
    ],
    "source": [
     "import numpy as np\n",
     "import pandas as pd\n",
     "from pandas import DataFrame\n",
     "def generate_parameters_I_L():\n",
     "    roundNum =3\n",
     "    result
=pd.DataFrame(columns=('a','b','c','d','k','m','mu','Cindy_Payoff','David_Payoff'))  \n",
     "    row = 0\n",
     "    for a in np.arange(0.1,1,0.1) : \n",
     "        for b in np.arange(0.1,1,0.1) : \n",
     "            for c in np.arange(0.1,1,0.1) : \n",
     "                for d in np.arange(0.1,1,0.1) : \n",
     "                    for k in np.arange(0.1,1,0.1) : \n",
     "                        for m in np.arange(0.1,1,0.1) : \n",
     "                            for mu in np.arange(0.1,1,0.1): \n",
     "                                a=round(a,roundNum)\n",
     "                                b=round(b,roundNum)\n",
     "                                c=round(c,roundNum)\n",
     "                                d=round(d,roundNum)\n",
     "                                k=round(k,roundNum)\n",
     "                                m=round(m,roundNum)\n",
     "                                mu=round(mu,roundNum)\n",
     "                                if d<k*(m*a+(1-m)*b)  and mu>=(1-(c/(k*d-b)))  :\n",
     "                                    Cindy_Payoff=1+k*(1-d)\n",
     "                                    David_Payoff=(1-mu)*(1-d+k)+mu*(1-d+k)\n",
     "                                    result.loc[row] =
[a,b,c,d,k,m,mu,Cindy_Payoff,David_Payoff] \n",
     "                            row += 1 \n",
     "    result.to_excel('I_L_data.xlsx',index=False)
```

```
\n",
    "generate_parameters_I_L()\n"
   ]
  },
  {
   "cell_type": "code",
   "execution_count": null,
   "metadata": {},
   "outputs": [
    {
     "name": "stderr",
     "output_type": "stream",
     "text": [
      "E:\\Program Files\\Install\\Python\\lib\\site-packages\\ipykernel_launcher.py:23:
RuntimeWarning: divide by zero encountered in double_scalars\n"
     ]
    }
   ],
   "source": [
    "import numpy as np\n",
    "import pandas as pd\n",
    "from pandas import DataFrame\n",
    "def generate_parameters_J_K():\n",
    "    roundNum = 3\n",
    "    #    print(\"a b c d k m\")\n",
    "    result
=pd.DataFrame(columns=('a','b','c','d','k','m','lamda','Cindy_Payoff','David_Payoff'))
\n",
    "    row = 0\n",
    "    for a in np.arange(0.1,1,0.1) : \n",
    "        for b in np.arange(0.1,1,0.1) : \n",
    "            for c in np.arange(0.1,1,0.1) : \n",
    "                for d in np.arange(0.1,1,0.1) : \n",
```

```
    "                    for k in np.arange(0.1,1,0.1) : \n",
    "                        for m in np.arange(0.1,1,0.1) : \n",
    "                            for lamda in np.arange(0.1,1,0.1): \n",
    "                                a=round(a,roundNum)\n",
    "                                b=round(b,roundNum)\n",
    "                                c=round(c,roundNum)\n",
    "                                d=round(d,roundNum)\n",
    "                                k=round(k,roundNum)\n",
    "                                m=round(m,roundNum)\n",
    "                                lamda=round(lamda,roundNum)\n",
    "                                if (d>k*(m*a+(1-m)*b) and lamda>=(1-(c/(a-k*d)))) :
\n",
    "                                    Cindy_Payoff=1-b+k+m*(b-a)\n",
    "                                    David_Payoff=1+k*(1-b)+m*k*(b-a)\n",
    "                                    \n",
    "                                    result.loc[row] =
[a,b,c,d,k,m,lamda,Cindy_Payoff,David_Payoff] \n",
    "                                row += 1                        \n",
    "\n",
    "    result.to_excel('J_K_data.xlsx',index=False) \n",
    "                                 \n",
    "\n",
    "generate_parameters_J_K()"
   ]
  },
  {
   "cell_type": "code",
   "execution_count": null,
   "metadata": {},
   "outputs": [],
   "source": [
    "import numpy as np\n",
    "import pandas as pd\n",
```

```
    "from pandas import DataFrame\n",
    "def generate_parameters_Hybrid_equilibrium():\n",
    "    roundNum = 3\n",
    "    result
=pd.DataFrame(columns=('a','b','c','d','k','m','lamda','mu','Cindy_Payoff','David_Payoff'
))  \n",
    "    row = 0\n",
    "    for a in np.arange(0.1,1,0.1) : \n",
    "        for b in np.arange(0.1,1,0.1) : \n",
    "            for c in np.arange(0.1,1,0.1) : \n",
    "                for d in np.arange(0.1,1,0.1) : \n",
    "                    for k in np.arange(0.1,1,0.1) : \n",
    "                        for m in np.arange(0.1,1,0.1): \n",
    "                            a=round(a,roundNum)\n",
    "                            b=round(b,roundNum)\n",
    "                            c=round(c,roundNum)\n",
    "                            d=round(d,roundNum)\n",
    "                            k=round(k,roundNum)\n",
    "                            m=round(m,roundNum)\n",
    "                            if ( a>d/k and d/k>b and b-k*d>c and d>k*(m*a+(1-m)*b)):
\n",
    "                                lamda = (k*(m*a+(1-m)*b)-d)/((1-m)*(k*b-d))\n",
    "                                mu  = c/(b-k*d)\n",
    "                                lamda=round(lamda,roundNum)\n",
    "                                mu=round(mu,roundNum)\n",
    "                                \n",
    "                                Cindy_Payoff = lamda*(1-b+k+m*(b-c-k*d)+1-b+k+m*(b-
a-c))/2+(1-lamda)*(1-b-c+k+m*(b-a)+1-c+k*(1-d))/2\n",
    "                                David_Payoff = mu*(1-k*b+k+m*(b*k-c*k-d)+1+k*(1-b)
+m*k*(b-a-c))/2+(1-mu)*(1+k*(1-b-c)+m*k*(b-a)+1-d+k*(1-c))/2\n",
    "                                Cindy_Payoff=round(Cindy_Payoff,roundNum)\n",
    "                                David_Payoff=round(David_Payoff,roundNum)\n",
    "                                result.loc[row] =
```

```
[a,b,c,d,k,m,lamda,mu,Cindy_Payoff,David_Payoff] \n",
    "                            row += 1 \n",
    "                                                        \n",
    "\n",
    "    result.to_excel('Hybrid_equilibrium_data.xlsx',index=False) \n",
    "    \n",
    "                                \n",
    "generate_parameters_Hybrid_equilibrium()"
   ]
  },
  {
   "cell_type": "code",
   "execution_count": null,
   "metadata": {},
   "outputs": [],
   "source": []
  }
 ],
 "metadata": {
  "kernelspec": {
   "display_name": "Python 3",
   "language": "python",
   "name": "python3"
  },
  "language_info": {
   "codemirror_mode": {
    "name": "ipython",
    "version": 3
   },
   "file_extension": ".py",
   "mimetype": "text/x-python",
   "name": "python",
   "nbconvert_exporter": "python",
```

```
   "pygments_lexer": "ipython3",
   "version": "3.7.4"
  }
 },
 "nbformat": 4,
 "nbformat_minor": 2
}
```

Policy.m

Matlab script starting from the next page (2 pages in total):

```
%%
clc;clear all;close all
A=xlsread('A_data.xlsx');
F=xlsread('F_data.xlsx');
Policy1=[A;F]
rowname={'a','b','c','d','k','m','Cindy_Payoff','David_Payoff'}
 A=[rowname; num2cell(A)] ;
 F=[rowname; num2cell(F)] ;
 Policy1=[rowname; num2cell( Policy1)] ;
xlswrite('ESS.xlsx',A,1)
xlswrite('ESS.xlsx',F,2)
xlswrite('Policy.xlsx',Policy1,1)

%%
clc;clear all;close all
I_L=xlsread('I_L_data.xlsx');
rowname={'a','b','c','d','k','m','lamda','Cindy_Payoff','David_Payoff'}
Policy3=[rowname;num2cell(I_L)] ;
I_L=[rowname; num2cell(I_L)] ;


xlswrite('ESS.xlsx',I_L,3)
xlswrite('Policy.xlsx',Policy3,2)

%%

clc;clear all;close all
J_K=xlsread('J_K_data.xlsx');
rowname={'a','b','c','d','k','m','miu','Cindy_Payoff','David_Payoff'}
```

```
Policy4=[rowname; num2cell(J_K)] ;
J_K=[rowname; num2cell(J_K)] ;
xlswrite('ESS.xlsx',J_K,4)
xlswrite('Policy.xlsx',Policy4,3)

%%
clc;clear all;close all
Hybrid=xlsread('Hybrid_equilibrium_data.xlsx');
rowname={'a','b','c','d','k','m','lamda','miu','Cindy_Payoff','David_Payoff'
}
 Policy5=[rowname; num2cell(Hybrid)] ;
Hybrid=[rowname; num2cell(Hybrid)] ;
xlswrite('ESS.xlsx',Hybrid,5)
xlswrite('Policy.xlsx',Policy5,4)

%%
clc;clear all;close all
e=actxserver('Excel.Application');
ewb=e.workbooks.Open('Policy.xlsx')
% ewb.worksheets.Item(1).Name='LOHC'
% ewb.worksheets.Item(2).Name='AC(merge)'
% ewb.worksheets.Item(3).Name='AO'
% ewb.worksheets.Item(4).Name='Hybrid'
% ewb.Close(false)
% e.Quit
```
